# Degradome sequencing-based identification of phasiRNAs biogenesis pathways in *Oryza sativa*

**DOI:** 10.1186/s12864-021-07406-7

**Published:** 2021-01-30

**Authors:** Lan Yu, Rongkai Guo, Yeqin Jiang, Xinghuo Ye, Zhihong Yang, Yijun Meng, Chaogang Shao

**Affiliations:** 1 College of Life Sciences, Huzhou University, NO.1 Xueshi Road, Huzhou, 313000 P.R. China; 2 Shanghai Institute of Plant Physiology and Ecology, Chinese Academy of Sciences, Shanghai, 200032 China; 3 College of Life and Environmental Sciences, Hangzhou Normal University, Xuelin Street 16#, Xiasha, Hangzhou, 310036 P. R. China

**Keywords:** *Oryza sativa*, Phased small interfering RNAs, Precursor, Degradome sequencing, Regulatory network

## Abstract

**Background:**

The microRNAs(miRNA)-derived secondary phased small interfering RNAs (phasiRNAs) participate in post-transcriptional gene silencing and play important roles in various bio-processes in plants. In rice, two miRNAs, miR2118 and miR2275, were mainly responsible for triggering of 21-nt and 24-nt phasiRNAs biogenesis, respectively. However, relative fewer phasiRNA biogenesis pathways have been discovered in rice compared to other plant species, which limits the comprehensive understanding of phasiRNA biogenesis and the miRNA-derived regulatory network.

**Results:**

In this study, we performed a systematical searching for phasiRNA biogenesis pathways in rice. As a result, five novel 21-nt phasiRNA biogenesis pathways and five novel 24-nt phasiRNA biogenesis pathways were identified. Further investigation of their regulatory function revealed that eleven novel phasiRNAs in 21-nt length recognized forty-one target genes. Most of these genes were involved in the growth and development of rice. In addition, five novel 24-nt phasiRNAs targeted to the promoter of an *OsCKI1* gene and thereafter resulted in higher level of methylation in panicle, which implied their regulatory function in transcription of *OsCKI1*,which acted as a regulator of rice development.

**Conclusions:**

These results substantially extended the information of phasiRNA biogenesis pathways and their regulatory function in rice.

**Supplementary Information:**

The online version contains supplementary material available at 10.1186/s12864-021-07406-7.

## Background

Small RNA-mediated RNA silencing is a conserved mechanism that regulates various bioprocesses in eukaryotes [1]. Two types of endogenous small RNAs, microRNAs (miRNAs) and small interfering RNAs (siRNAs), are highly abundant in plants.

The biogenesis of a miRNA in plants begins with the transcription of a primary miRNA (pri-miRNA). Next, an RNase III family of DICER-LIKE (DCL) enzyme, usually DCL1, sequentially processes the pri-miRNA into a precursor (pre-miRNA) and further cut into a miRNA/miRNA* short duplex with the help of HYPONASTIC LEAVES 1 (HYL1) and SERRATE (SE). This results in one strand (miRNA*) of the short duplex is degraded and the mature miRNA strand is incorporated into the RNA-induced silencing complex (RISC), which is highly complementary to the target gene and subsequently leads to the cleavage of the target mRNA followed by its degradation in plants [2]. Recently, research indicated the critical roles of miRNAs in various biological processes in plants, such as growth and development, stress response and plant metabolism [3, 4]. For example, OsmiR393a and OsmiR393b regulated rice primary root elongation and adventitious roots number via auxin signaling pathway [5]. The miR398 directly linked to the *Arabidopsis* stress regulatory networks such as oxidative stress,water deficit, salt stress, etc. [6].

In terms of siRNAs, their biogenesis could be triggered either endogenously by its genetic events or exogenously causes, such as virus infection or transgenic operation [7]. In contrast to miRNA, the precursor of siRNAs are usually long and double-stranded [7]. Recent years, researchers found the biogenesis of some siRNAs are “triggered” by miRNAs-mediated cleavage. Fragments resulted from mRNA cleavage are typically subjected to rapid degradation. However, a small proportion of the fragments will survive and subsequently be processed into double-stranded RNA (dsRNA) by RNA-dependent RNA polymerase 6(RDR6) with the aid of Suppressor of Gene Silencing 3 (SGS3). These double-stranded fragments will further be cleaved by Dicer-like (DCL) proteins in different phased manners to produce a series of 21- or 24-nt siRNAs, termed phased small interfering RNAs (phasiRNAs) [8].

SiRNAs in 21-nt length regulates gene expression by cleaving complementary transcripts the same as miRNA-mediated cleavage in plant. The best-characterized phasiRNAs are *TAS loci*-derived 21-nt *trans*-acting siRNAs (tasiRNAs) in *Arabidopsis*. Research discovered that miR173 targeted to *TAS1* and *TAS2* and resulted in the production of tasiRNAs. Interestingly, some of these tasiRNAs continued to recognize target transcripts to produce tertiary phasiRNAs [9]. The *TAS1*- and *TAS2*-derived tasiRNAs were involved in regulation of stress responses, such as improvement of thermotolerance [10], maintaining the normal morphogenesis of flowers in plants under drought stress conditions [11]. The biogenesis of *TAS3-derived* tasiRNAs were triggered by the miR390 recognition [12]. These tasiRNAs targeted to *ARF* family members which regulates various biological processes, including embryo development, thermotolerance developmental transitions, leaf morphology, flower and root architecture and stress responses [13, 14]. Besides, report showed *TAS4*-derived tasiRNAs induced by miR828 regulated anthocyanin biogenesis via repression of *MYB* genes [15]. For siRNAs in 24-nt length, researches revealed that they were key players in triggering of RNA-directed DNA methylation (RdDM) [16], which is the major small RNA-mediated epigenetic pathway that causes transposable element repression and transcriptional gene silencing (TGS) in plants [17]. For example, recent research discovered that the distribution of 24-nt siRNAs differs in rice gametes (sperm and egg), as well as from vegetative tissues, which further suggest a major difference in reprogramming of their genomes prior to fertilization [18].

Different algorithm and software tools have been employed not only in mining the novel miRNA-phasiRNA pathways, but in exploring the miRNAs’ extended regulatory networks [19]. Current research discovered two miRNAs, miR2118 and miR2275, were mainly responsible for the triggering of 21-nt and 24-nt phasiRNAs biogenesis, respectively [20]. And subsequent reports were then focused on the investigation of miR2118-phasiRNA and miR2275-phasiRNA biogenesis pathways and their biological functions [21, 22]. To our knowledge, about 56 phasiRNA precursors (PHAS loci) have been identified in rice. For PHAS loci in other economic crops, approximately 261 PHAS loci in *Zea mays* (maize), 916 PHAS loci in *Setariaitalica* (foxtail millet), 201 PHAS loci in *Solanumtuberosum* (potato), and 123 in *Solanumlycopersicum*(tomato) have been discovered, respectively [23]. Besides, in addition to those non-coding regions in genome, protein-coding genes could also be the PHAS loci in plants [23, 24], which implies a more complicated mechanism of plant phasiRNA biogenesis.

Due to the biological significance of phaiRNAs, mining of novel miRNA-phasiRNA pathways as well as functional cascade amplification have attracted wide attention. As an important economic crop, investigating novel phasiRNA pathways will not only benefit our understanding in post-transcriptional regulations in this organism, but also could be used as references across economic crops.

Previously, we discovered lots of siRNAs in a three-week-old seedling sample by using the corresponding sRNA high-throughput screening (HTS) datasets. This inspired us that some of them might be phasiRNAs. Here, we continued to use our previously developed approach [25] for systematically mining of phasiRNA biogenesis pathways with these sRNA HTS datasets. In addition, considering some phasiRNAs expression might be tissue specific or stress dependent, we collected comparable sRNA HTS data sets published elsewhere using tissue-specific rice samples, which cultured under normal (control) or stress condition. The targets of novel phasiRNAs were further predicted and verified in order to provide substantial information of miRNA/sRNA-phasiRNA regulatory network in rice.

## Results

### Identification of novel phasiRNA biogenesis pathways in *Oryza sativa*

The sRNA HTS datasets from different rice samples were employed as inputs and rice cDNA sequences as alignment reference for searching PHAS loci capable of producing 21-nt or 24-nt phasiRNAs. As a result, fourteen 21-nt and nineteen 24-nt PHAS loci candidates passed through the filtering procedures as well as the corresponding searching of sRNA triggers for phasiRNA production (Additional file 1:Table S1, Additional file 2: Table S2). Recent reports discovered that processing of 21-nt phasiRNAs mainly depends on OsDCL4, and OsDCL3 is required for biogenesis of 24-nt phasiRNAs in rice [20]. Therefore, we evaluated the abundance of 21-nt and 24-nt phasiRNAs generated from potential PHAS loci candidates by comparing the wild-type (wt) with *osdcl4* knockdown mutant (*osdcl4–1*) [26] (for 21-nt phasiRNAs) and *osdcl3* knockdown mutant (*osdcl3–1*) [20] (for 24-nt phasiRNAs), respectively.

**Fig. 1:**
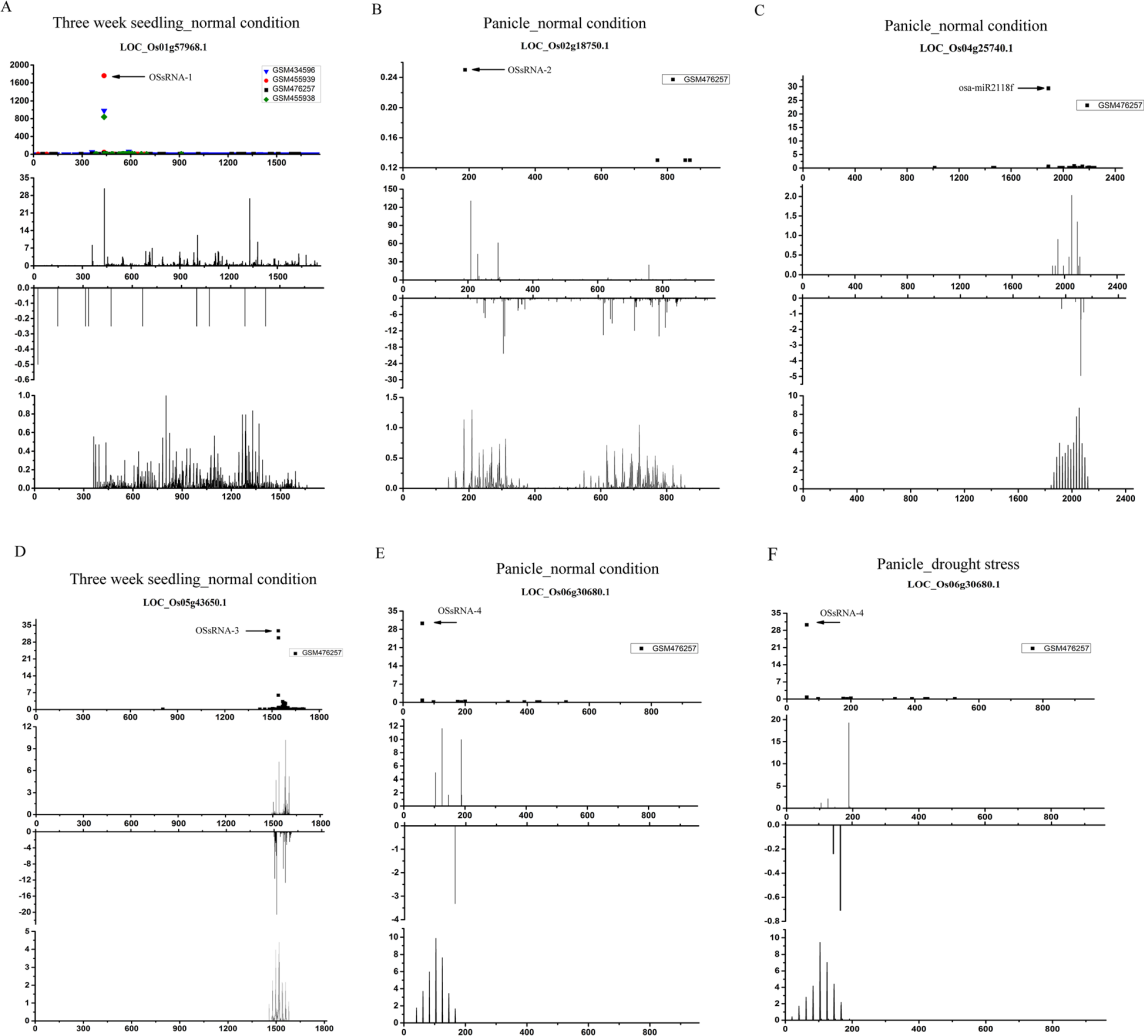
Identification of novel 21-nt phasiRNAs biogenesis pathways in *Oryza sativa*. **a** OSsRNA-1 induced phasiRNAs generation from the transcript of *LOC_Os01g57968.1* in seedling, **b** OSsRNA-2 induced phasiRNAs generation from the transcript of *LOC_Os02g18750.1* in panicle, **c** osa-miR2118f induced phasiRNAs generation form the transcript of *LOC_Os04g25740.1* in panicle, **d** OSsRNA-3 induced phasiRNAs generation from the transcript of *LOC_Os05g43650.1* in seedling, **e** OSsRNA-4 induced phasiRNAs generation from the transcript of *LOC_Os06g30680.1* in panicle, and **f** OSsRNA-4 induced phasiRNAs generation from the transcript of *LOC_Os06g30680.1* in panicle (drought stress). For each graph, degradome supported cleavage signature on PHAS loci were profiled above, four high throughput degradome sequencing datasets (GSM434596, GSM455938, GSM455939 and GSM476257 which were represented by triangle, diamond, circle and square with different colors, respectively) were employed for scanning the sRNA triggers’ cleavage sites, which were marked by black arrows. The x axis represents the position on PHAS loci and the y axis represents the signature abundance. The abundance of 21-nt phasiRNAs which generated from the sense and antisense strand of PHAS loci in different samples were evaluated and profiled in middle images, the x axis represents the position on PHAS loci and the y axis represents the phasiRNA abundance. The phasing score of 21-nt *PHAS* windows were profiled at bottom, the x axis represents the position on PHAS loci and the y axis represents the phasing score

**Fig. 2:**
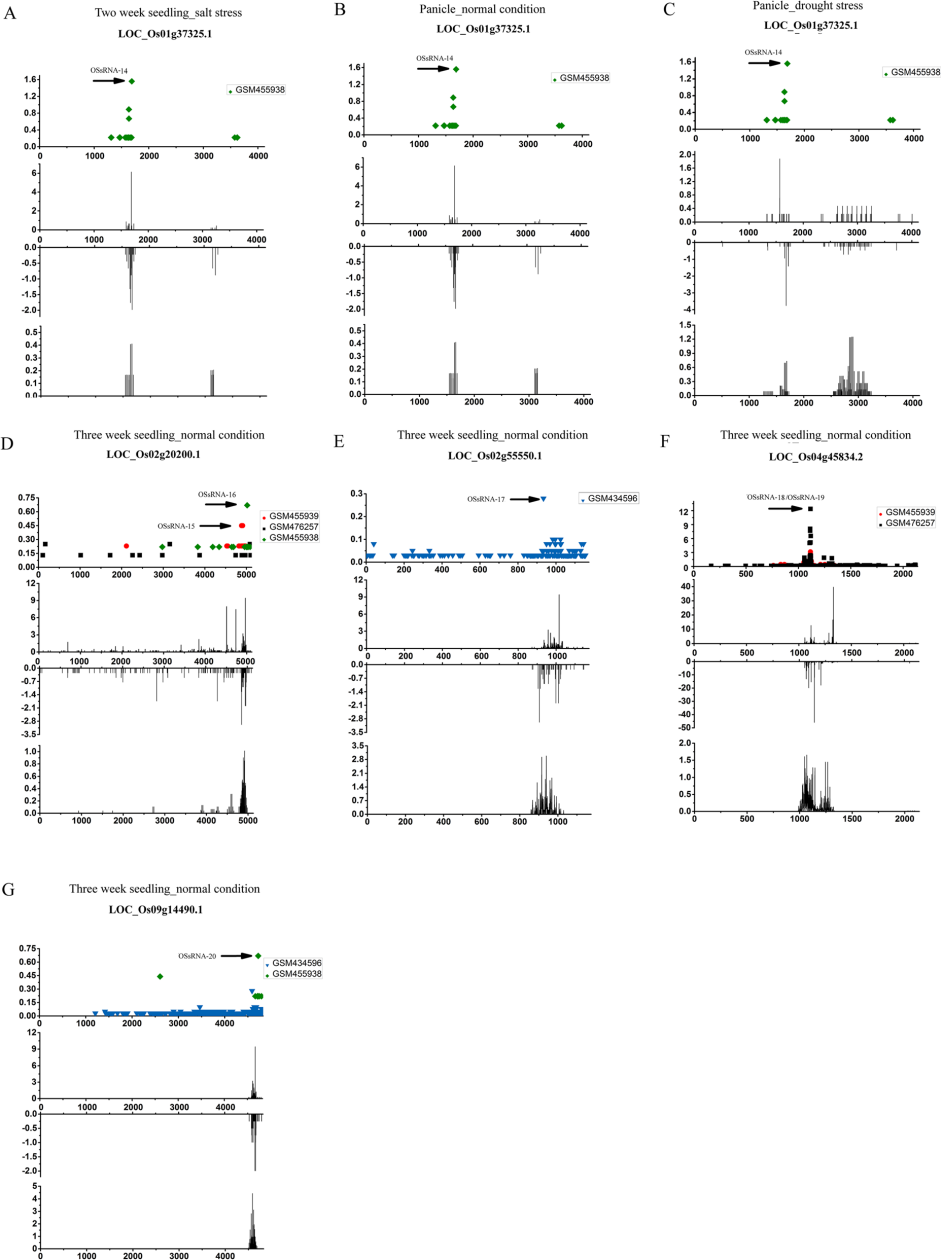
Identification of novel 24-nt phasiRNAs biogenesis pathways in *Oryza sativa*. **a** OSsRNA-14 induced phasiRNAs generation from the transcript of *LOC_Os01g37325.1* in seedling (salt stress). **b** OSsRNA-14 induced phasiRNAs generation from the transcript of *LOC_Os01g37325.1* in panicle. **c** OSsRNA-14 induced phasiRNAs generation from the transcript of *LOC_Os01g37325.1* in panicle (drought stess). **d** OSsRNA-15 and OSsRNA-16 induced phasiRNAs generation from the transcript of *LOC_Os02g20200.1* in seedling. **e** OSsRNA-17 induced phasiRNAs generation from the transcript of *LOC_Os02g55550.1* in seedling. **f** OSsRNA-18 or OSsRNA-19 induced phasiRNAs generation from the transcript of *LOC_Os04g45834.2* in seedling. **g** OSsRNA-20 induced phasiRNAs generation from the transcript of *LOC_Os09g14490.1* in seedling. For each graph, degradome supported cleavage signature on PHAS loci were profiled above, four high throughput degradome sequencing datasets (GSM434596, GSM455938, GSM455939 and GSM476257 which were represented by triangle, diamond, circle and square with different colors, respectively) were employed for scanning the sRNA triggers’ cleavage sites, which were marked by black arrows. The x axis represents the position on PHAS loci and the y axis represents the signature abundance. The abundance of 24-nt phasiRNAs which generated from the sense and antisense strand of PHAS loci in different samples were evaluated and profiled in middle images, the x axis represents the position on PHAS loci and the y axis represents the phasiRNA abundance. The phasing score of 24-nt *PHAS* windows were profiled at bottom, the x axis represents the position on PHAS loci and the y axis represents the phasing score

As a result, five novel 21-nt PHAS loci and five novel 24-nt PHA loci along with their corresponding miRNA/sRNA triggers were identified (Table 1). As shown in Fig. 1 and Fig. 2, the miRNA/sRNA triggers-mediated cleavages in target PHAS loci were detected by at least one degradome sequencing dataset. Indeed, each cleavage site was close to the flank of phasiRNA production region as indicated by the relative abundances of phasiRNAs (middle panel), which suggested these sites were primary registers for phasing process. Additionally, the abundance of phasiRNAs generated from these newly found 21-nt and 24-nt PHAS loci in wild type were relatively higher than that in *osdcl4–1* mutant and *osdcl3–1* mutant, respectively. This indicated that the 21-nt and 24-nt phasiRNA productions are OsDCL4- and OsDCL3 dependent, respectively (Additional file 3: Figure S1 and Additional file 4: Figure S2). Taken together, these results demonstrated that these newly found PHAS loci fit the profiles of canonical phasiRNA precursors [8, 20].

**Table 1:** Novel PHAS loci in *Oryza sativa*

Gene ID of the PHAS loci	Gene annotation	PhasiRNA production region	sRNA trigger ID	sRNA trigger sequence	Binding sites of sRNA trigger on gene of PHAS loci	Cleavage sites discovered by degradome on gene of PHAS loci
21-nt PHAS loci						
LOC_Os01g57968.1	expressed protein	361–1765	OSsRNA-1	GCUUUUUUGAACUUUUUCAUU	424–444	435
LOC_Os02g18750.1	expressed protein	188–920	OSsRNA-2	UUUUUUGGCAUUCUGUAACUUG	176–197	188
LOC_Os04g25740.1	expressed protein	1908–2159	osa-miR2118f	UUCCUGAUGCCUCCCAUUCCUA	1875–1896	1887
LOC_Os05g43650.1	expressed protein	1494–1620	OSsRNA-3	GAUUCAUUAACUUCAAUAUGAA	1528–1549	1540
LOC_Os06g30680.1	WD domain, G-beta repeat domain containing protein	62–208	OSsRNA-4	UUCCUGGAGCCGCUCAUUCCAU	50–71	62
24-nt PHAS loci						
LOC_Os01g37325.1	retrotransposon protein	1565–1760	OSsRNA-14	AAAAGUAGAUGGAUGCGGAGAC	1676–1697	1688
LOC_Os02g20200.1	retrotransposon protein	4856–5052	OSsRNA-15	UAGAUGCUGUCCUGAAAAGGUG	4873–4894	4885
			OSsRNA-16	AGCCAUGCUAGUCUAAGAGGG	5007–5027	5018
LOC_Os02g55550.1	F-box/LRR-repeat protein 14	905–1101	OSsRNA-17	UAGAUGCUGUCCUGAAAAGGUG	922–943	934
LOC_Os04g45834.2	DUF584 domain containing protein	1051–1307	OSsRNA-18/ OSsRNA-19	UUAAUAUUUAUAAUUAGUGUCU/ UUAAUAUUUAUAAUUAAUGUCC	1103–1124	1115
LOC_Os09g14490.1	TIR-NBS type disease resistance protein	4585–4757	OSsRNA-20	UAGAUGCUGUCCUGAAAAGGUG	4578–4599	4590

Previously, we found lots of sRNAs generated in three-week-old seedling tissues (see details about the information of GEO number of dataset and culture condition of plants in Additional file 5: Table S3**)**. Here, we tested whether these sRNA are phasiRNAs by using our mining method. As expected, the phasiRNAs generated from two novel 21-nt PHAS loci (*LOC_Os01g57968.1*and *LOC_Os05g43650.1*) and four novel 24-nt PHAS loci (*LOC_Os02g20200.1*, *LOC_Os02g55550.1*, *LOC_Os04g45834.2* and *LOC_Os09g14490.1*) were identified in three-week-old seedling tissues (Table 1).

Since the triggering of phasiRNAs are sometimes tissue specific and stress dependent, a serial of sRNA HTS datasets of different rice samples were employed for mining novel PHAS loci in different tissues and stress conditions (see details about the information of GEO number of datasets, culture and treatment conditions of plants in Additional file 5: Table S3**)**. As shown in Fig. 1, transcripts of 21-nt PHAS loci, *LOC_Os02g18750.1* and *LOC_Os04g25740.1*, were able to produce 21-nt phasiRNAs in panicle under normal condition. *LOC_Os06g30680.1*-derived 21-nt phasiRNAs and *LOC_Os01g37325.1*-derived 24-nt phasiRNAs were detected in panicle under both drought and normal condition.

According to the gene annotation, *LOC_Os01g57968.1*, *LOC_Os02g18750.1*, *LOC_Os04g25740.1* and *LOC_Os05g43650.1* encode proteins with unknown function and *LOC_Os06g30680.1* encodes a WD domain, G-beta repeat domain containing protein. *LOC_Os01g37325.1* and *LOC_Os02g20200.1* encode two retrotransposon genes, *LOC_Os02g55550.1* encodes an F-box/LRR-repeat protein, *LOC_Os04g45834.2* encodes a protein with DUF584 domain, and *LOC_Os09g14490.1* encodes a TIR-NBS type disease resistance protein.

Taken together, these protein-coding genes acted as PHAS loci in different tissues and stress conditions suggested these coding sequences were regulated at post-transcriptional level in response to different stages of growth and stress conditions.

In consistent to previous discovery, two known 21-nt PHAS loci (*LOC_Os12g42380.1* and *LOC_Os12g42390.1*) were also uncovered by our screening procedure (Additional file 1: Table S1), which have been identified as two parts of a long non-coding RNAs [27]. *LOC_Os12g42380.1*-derived phasiRNAs were detected in both seedling and panicle under normal, drought and salinity stress conditions. Yet they were only detected in shoot under salinity stress. *LOC_Os12g42390.1*-derived phasiRNAs were detected in shoot under normal condition, and panicle in drought. These results implied there are three alternative phasiRNA production regions within their lncRNA PHAS loci, and therefore the capability of phasiRNA production might vary in different development stages and stress conditions.

To note, to our knowledge, for all these newly found PHAS loci, only the biogenesis of *LOC_Os04g25740.2*-derived phasiRNAs were triggered by a known miRNA, miR2118f. The rest of them were first-time discovered, and were recognized by novel sRNAs (Table 1), which suggested these phasiRNA biogenesis pathways are not belong to the miR2118 or miR2257 mediated regulatory networks.

### Analysis of the regulatory function of novel phasiRNAs generated from 21-nt PHAS loci

**Fig. 3:**
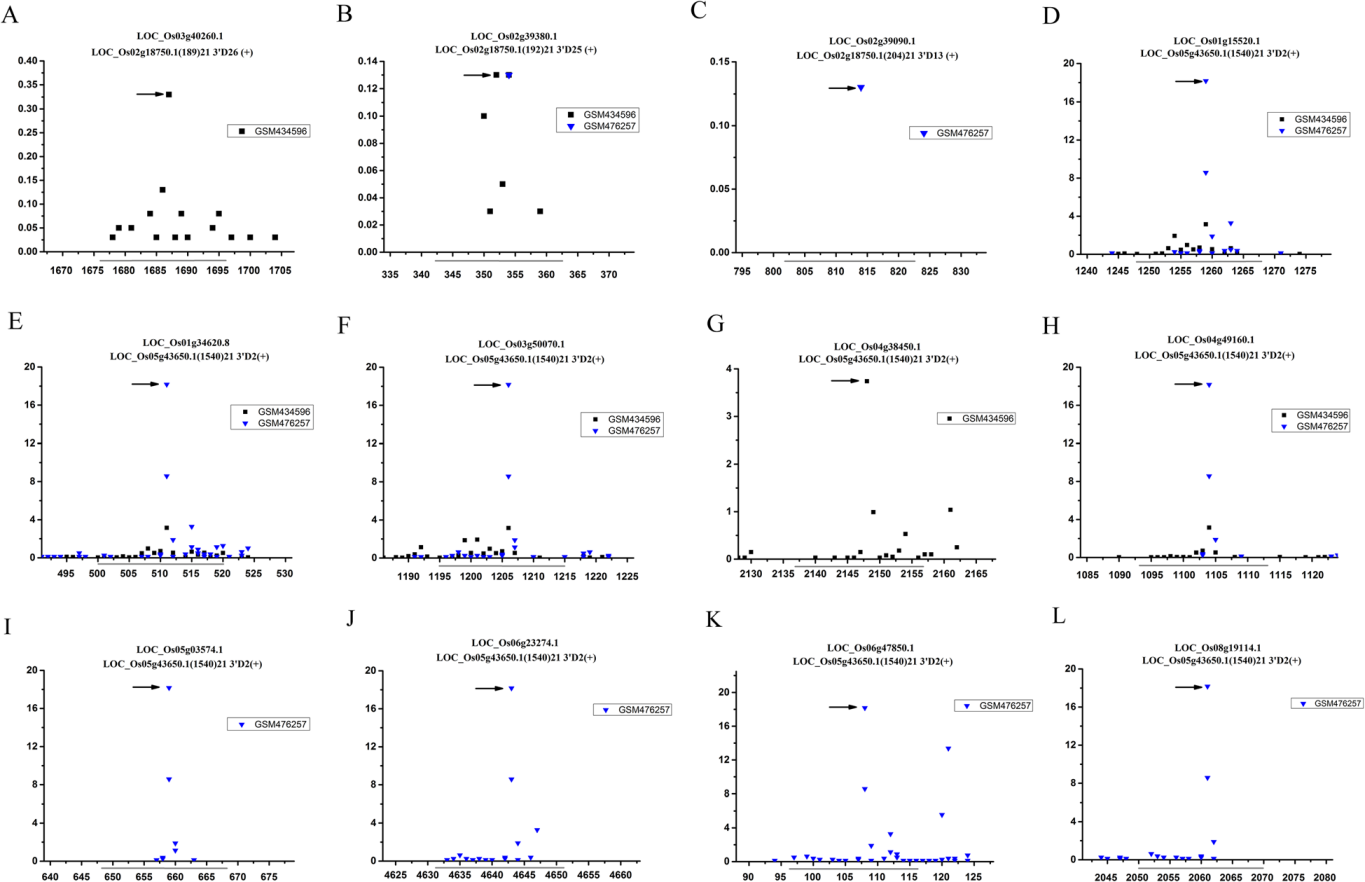
Examples of degradome sequencing-based validation of the phasiRNA-target interactions. Two libraries of degradome sequencing data libraries (GSM434596 and GSM476257) were recruited for T-plot profiling. The IDs of the target transcripts and the corresponding phasiRNAs generated from the transcript of *LOC_Os02g18750.1*and *LOC_Os05g43650.1* are listed on the top. The *y* axis measure the normalized reads (in RMP, reads per million) of the degradome signals, and the *x* axis represent the position of the cleavage signals on the target transcripts. The binding sites of the phasiRNA on their target transcripts were denoted by gray horizontal lines, and the dominant cleavage signals were marked by black arrows

The tasiRNAs are those 21-nt phasiRNAs with *trans-*regulatory function by cleaving target mRNAs in plant. In order to identify novel tasiRNAs generated from the newly found 21-nt PHAS loci, all the 21-nt phasiRNAs were systematically “predicted” based on modified tasiRNA biogenesis model [28]. All of detectable phasiRNAs were then employed for target prediction based on miRU algorithm and verified by using degradome-based HTS data (see details in “methods”). The results indicated ten novel tasiRNAs were generated from three newly found 21 nt PHAS loci (*LOC_Os02g18750.1*, *LOC_Os05g43650.1* and *LOC_Os06g30680.1*). These tasiRNAs mediated forty sRNA-target interactions (Table 2, Fig. 3, Additional file 6: Figure S3). Among these targets, *LOC_Os02g39380.1* played important roles in plant cellular signaling cascades [29]. *LOC_Os01g34620.8*, *LOC_Os02g52900.2*, *LOC_04g39600.1*, *LOC_08g40440.1*, *LOC_Os6g23274.1*, *LOC_Os06g47850.1*, *LOC_11g41860.1,LOC_11g41860.2* and *LOC_Os05g46580.1* were involved in plant growth and development [30–35]. *LOC_Os09g12230.1*, *LOC_Os04g38450.1* and *LOC_Os04g49160.1* were related to plant defense and stress response [36–38].

**Table 2:** Targets of novel tasiRNAs in *Oryza sativa*

TaisRNA ID	tasiRNA sequence	Targets	Target annotation	miRU start-ending	taisRNA mediated cutsites
LOC_Os02g18750.1(189)21 3’D26 (+)	UGUGCCACGUCAACACCACCA	LOC_Os03g40260.1	Regulator of chromosome condensation domain containing protein	1676–1696	1687
LOC_Os02g18750.1(192)21 3’D25 (+)	GCGCCACUGCCGUCGACGUGU	LOC_Os02g39380.1	OsCML17 - Calmodulin-related calcium sensor protein	343–363	354
LOC_Os02g18750.1(204)21 3’D13 (+)	UCGACUUCGCCGCCUCGGCGC	LOC_Os02g39090.1	expressed protein	802–823	814
LOC_Os05g43650.1(1540)21 3’D2(+)	UCAAUAUGAAUGUGGAAAAUG	LOC_Os01g15520.1	expressed protein	1248–1268	1259
		LOC_Os01g34620.8	OsGrx_S15.1 - glutaredoxin subgroup II	500–520	511
		LOC_Os03g50070.1	DUF1295 domain containing protein	1195–1215	1206
		LOC_Os04g38450.1	gamma-glutamyltranspeptidase 1 precursor	2137–2157	2148
		LOC_Os04g49160.1	zinc finger, C3HC4 type domain containing protein	1093–1113	1104
		LOC_Os05g03574.1	expressed protein	648–668	659
		LOC_Os06g23274.1	zinc finger, C3HC4 type, domain containing protein	4632–4652	4643
		LOC_Os06g47850.1	zinc finger family protein	97–117	108
		LOC_Os08g19114.1	expressed protein	2050–2070	2061
		LOC_Os08g40440.1	dihydroflavonol-4-reductase	1315–1335	1326
		LOC_Os09g12230.1	ubiquitin-conjugating enzyme	1021–1041	1032
		LOC_Os09g27500.1	cytochrome P450	1714–1734	1725
		LOC_Os11g41860.1	OsFBX429 - F-box domain containing protein	1030–1050	1041
		LOC_Os11g41860.2	OsFBX429 - F-box domain containing protein	973–993	984
		LOC_Os12g12950.1	expressed protein	1071–1091	1082
LOC_Os05g43650.1(1540)21 3’D2(−)	UUUUCCACAUUCAUAUUGAUG	LOC_Os02g45650.1	peptidase	1760–1780	1771
LOC_Os05g43650.1(1542)21 3’D1(+)	AAUGAAUCUAGACAUAUAUAU	LOC_Os02g05810.1	expressed protein	1330–1350	1341
		LOC_Os02g05810.2	expressed protein	1324–1344	1335
		LOC_Os02g52900.2	glutaredoxin 2	2034–2054	2045
		LOC_Os02g53000.2	lysM domain-containing GPI-anchored protein precursor	1340–1360	1351
		LOC_Os04g44590.1	expressed protein	651–671	662
		LOC_Os04g44590.5	expressed protein	445–465	456
		LOC_Os05g41190.1	expressed protein	1026–1046	1037
		LOC_Os05g41190.2	expressed protein	1082–1102	1093
		LOC_Os05g51140.1	expressed protein	929–949	940
		LOC_Os05g51140.2	expressed protein	1586–1606	1597
		LOC_Os09g33930.1	farnesyltransferase/geranylgeranyltransferase type-1 subunitalph	1457–1477	1468
		LOC_Os09g33930.2	farnesyltransferase/geranylgeranyltransferase type-1 subunitalph	1454–1474	1465
		LOC_Os09g33930.3	farnesyltransferase/geranylgeranyltransferase type-1 subunitalph	1740–1760	1751
		LOC_Os09g33930.4	farnesyltransferase/geranylgeranyltransferase type-1 subunitalph	1453–1473	1464
		LOC_Os09g33930.5	farnesyltransferase/geranylgeranyltransferase type-1 subunitalph	1375–1395	1386
		LOC_Os12g37510.1	UDP-glucoronosyl and UDP-glucosyltransferase domain containing	1584–1604	1595
LOC_Os05g43650.1(1543)21 3’D2(−)	GCAUUUUCCACAUUCAUAUUG	LOC_Os02g48390.1	phosphoribosyltransferase	1758–1778	1769
LOC_Os05g43650.1(1543)21 3’D3(−)	UUCACAAUGUAAGUCAUUUUA	LOC_Os04g39600.1	fasciclin domain containing protein	1020–1040	1031
		LOC_Os07g01130.1	pentatricopeptide containing protein	4240–4260	4251
LOC_Os05g43650.1(1543)21 3’D1(+)	AUGAAUCUAGACAUAUAUAUC	LOC_Os12g40920.1	bZIP transcription factor domain containing protein	1312–1332	1323
LOC_Os06g30680.1(62)21 3′ D2(+)	CAUGGACAACUUCCUGCACAG	LOC_Os05g46580.1	polyprenylsynthetase	1365–1385	1376
LOC_Os12g42380.1(414)21 5’D7(+)	UUUCUUCCAAGAGAGAGUAAG	LOC_Os07g47700.1	NAD dependent epimerase/dehydratase family domain containing protein	1753–1773	1764

**Fig. 4:**
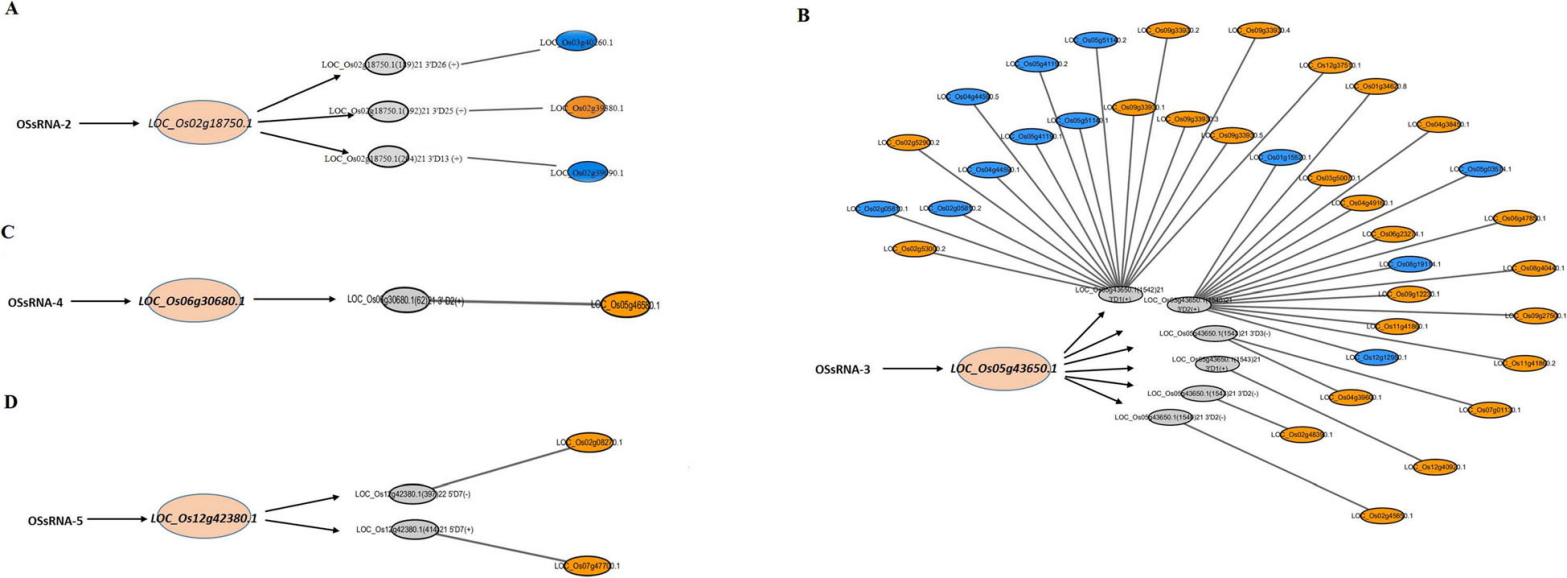
The regulatory networks of phasiRNA pathways in *Oryza sativa*. The OSsRNA-2- *LOC_Os02g18750.1-phasiRNA* (**a**), OSsRNA-3- *LOC_Os05g43650.1-*phasiRNA (**b**), OSsRNA-4- *LOC_Os06g30680.1-*phasiRNA (**c**) and OSsRNA-5- *LOC_Os12g42380.1-phasiRNA* (**d**) regulatory network were constructed by Cytoscape based on the validated phasiRNAs and their targets. The phasiRNAs are the gray nodes, the orange nodes represent the targets involving plant development, stress response, disease resistance or signaling transport. The blue nodes represent the expressed proteins with unknown functions

Although the transcript of *LOC_Os12g42380.1* has been identified as part of an lncRNA phasiRNA precursor [27], one novel *LOC_Os12g42380.1*-derived tasiRNA was found based on our revised tasiRNA biogenesis model [28]. LOC_Os12g42380.1 (414)21 5’D7(+) targeted to a NAD-dependent epimerase/dehydratase gene (*LOC_Os07g47700.1*) (Table 1, Additional file 6: Figure S3) suggested it might be involved in regulation of plant growth, development and environmental stress [39, 40]. Taken together, these results suggested the OSsRNA-2-*LOC_Os02g18750.1*-phasiRNA, OSsRNA-3-*LOC_Os05g43650.1*-phasiRNA, OSsRNA-4-L*OC_Os06g30680.1*-phasiRNA and OSsRNA-5-*LOC_Os12g42380.1*-phasiRNA pathways might play crucial regulatory roles in rice growth, development and stress response. In addition, the regulatory networks of the phasiRNA pathways mentioned above were constructed based on the target information (Fig. 4).

### Analysis of the RNA directed DNA methylation (RdDM) regulated promoters of novel 24-nt phasiRNAs

**Fig. 5:**
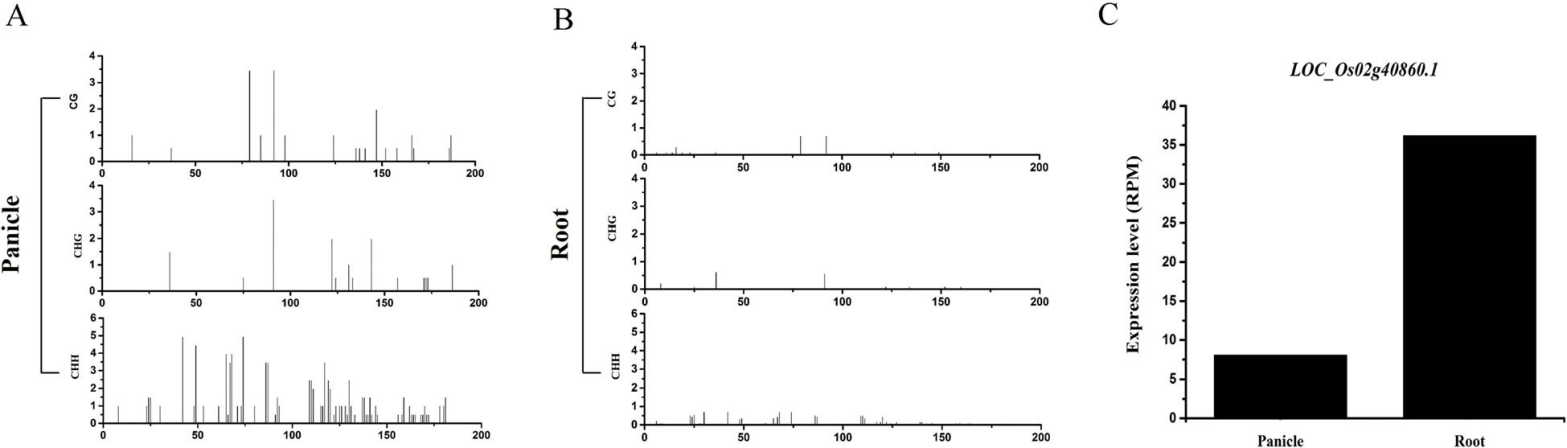
DNA methylation status and expression analysis of target promoter. DNA methylation by CG, CHG and CHH context at the promoter of *LOC_Os02g40860.1*in panicle (**a**) and root(**b**) were analyzed and profiled. X-axis represents the position on promoter sequence and Y-axis represents the abundance of CG, CHG or CHH. The expression level of *LOC_Os02g40860.1* in panicle and root were also showed in a bar chart(**c**)

RdDM is an important regulatory event with regards to repressive epigenetic modification which triggers transcriptional gene silencing. In order to analysis the novel 24-nt phasiRNA mediated RdDM in rice, we focused on all the known promoter sequences for scanning the target sites of novel phasiRNAs generated from the newly found five 24-nt PHAS loci. The result indicated a promoter of *LOC_Os02g40860.1* gene was targeted by five *LOC_Os01g37325.1*-derived phasiRNAs (Table 3). Since *LOC_Os01g37325.1*-derived phasiRNAs were detected in panicle rather than in root tissue (Fig. 2), we used the bisulfite-seq and RNA-seq datasets [41] of rice panicle and root for identification of *LOC_Os01g37325.1*-derived phasiRNAs-mediated DNA methylation intarget promoter and their role in transcriptional repression of target gene (*LOC_Os02g40860.1*). It was reported that CG and CHG methylation contexts are maintained by DNA methyltransferases and histone modifications, while CHH methylation was associated with 24-nt siRNA guided RdDM [16]. We discovered the CHH methylation status of promoter was relative higher in panicle than in root (Fig. 5). In addition, the expression level of *LOC_Os02g40860.1* was relatively lower in panicle than in root. These results implied a methylation mediated transcriptional silencing of the promoter of *LOC_Os02g40860.1*.

**Table 3:** The target promoter of LOC_Os01g37325.1-derived phasiRNAs

24-nt phasiRNAs_ID	PhasiRNAs_sequences	Binding_sites_ on_promoter	Prmoter_location	Target_genes	Target annotation
LOC_Os01g37325.1(1684) 24 5’D12(+)	AUCAUGACUUGGGUAUUACGUUUC	111–134	chr2_24766608–24,766,807	LOC_Os02g40860.1	Casein kinase I1 (CKI1)
LOC_Os01g37325.1(1684) 24 5’D10(+)	AGUCCUGGUUUGAUAAGAUUGUAA	63–86			
LOC_Os01g37325.1(1684) 24 5’D9(+)	AGUAGAUUUAGGAAACCGAUACCG	39–62			
LOC_Os01g37325.1(1665) 24 5’D13(+)	ACUAGUUAUAGGGGAUAACUUAUA	154–177			
LOC_Os01g37325.1(1665) 24 5’D11(+)	GACUUGGGUAUUACGUUUCCCUGU	106–129

For *LOC_Os02g40860.1*, it encodes a Casein kinase I1 (OsCKI1) protein belongs to the CKIs protein family, which are highly conserved in eukaryotes. They are involved in a variety of important biological events since they have a wide substrate specificity in vitro [42]. Taken together, we speculated that the OSsRNA-14-*LOC_Os01g37325.1*-phasiRNA pathway might play crucial roles for rice seedling and panicle development.

## Discussion

In recent years, researches on *Oryza sativa* have shown that 21- or 24-nt sRNAs distribute to genomic clusters [43]. To date, dozens of PHAS loci have been discovered in rice [23]. However, two miRNAs, miR2118 and miR2275 are mainly responsible for the triggering of 21-nt or 24-nt phasiRNAs biogenesis from these PHAS loci.

Considering there are rich sources for miRNA/sRNA-phasiRNA pathways in other plant species, we conceived that the miRNA-phasiRNA pathways have not been fully discovered in rice either. Therefore, it is worthy of continuing the mining for better understanding the mechanism of phasiRNA biogenesis and the miRNA-derived regulatory network. In our previous work, we found plenty of sRNAs with unknown function and origin from a sRNA HTS data set of three-week-old seedling tissue, and speculated some of them were phasiRNAs with regulatory functions. In this study, we performed a systematically searching of novel PHAS loci from rice cDNA by utilizing the same seedling dataset with our previous established mining approach [25].

As we expected, two novel 21-nt phasiRNA biogenesis pathways (OSsRNA-2-*LOC_Os01g57968.1*-phasiRNA and OSsRNA-3-*LOC_Os05g43650.1*-phasiRNA pathway) and four novel 24-nt phasiRNA biogenesis pathways (OSsRNA-15/OSsRNA-16-*LOC_Os02g20200.1*-phasiRNA, OSsRNA-17-*LOC_Os02g55550.1*-phasiRNA, OSsRNA-18/OSsRNA-19-*LOC_Os04g45834.2*-phasiRNA and OSsRNA-20-*LOC_Os09g14490.1*- phasiRNA pathway) were discovered. In addition, since the phasiRNAs are involved in regulation of plant growth and development, stress responses, we integrated a serial of sRNA HTS datasets from different tissues (including two-week-old seedling samples) under normal and stress conditions. As a result, three novel 21-nt phasiRNA biogenesis pathways (OSsRNA-2-*LOC_Os02g18750.1*-phasiRNA, osa-miR2118f-*LOC_Os04g25740.1*-phasiRNA and OSsRNA-4-*LOC_Os06g30680.1*-phasiRNA pathway) and one novel 24-nt phasiRNA biogenesis pathway (OSsRNA-2-LOC_Os01g37325.1-phasiRNA) were discovered. These results substantially extend the knowledge in phasiRNA biogenesis pathways in rice. However, the six novel phasiRNAs biogenesis pathways that we discovered in three-week-old seedling were undetected in two-week-old seedling samples, which might be caused by the low expression level of phasiRNAs generated from these pathways in younger seedlings.

The novel 21-nt PHAS loci, *LOC_Os05g43650.1*, is a miniature inverted-repeat transposable element (MITE) gene [44]. Also, with regards to two 24-nt PHAS loci, *LOC_Os01g37325.1* and *LOC_Os02g20200.1*, they are two retrotransposon genes. These indicated that the transcripts of transponsons and retrotransponsons are capable of producing secondary siRNAs, which is consistent with the same phenomenon reported by Creaseyet al. in *Arabidopsis* [45, 46].

According to the target information of phasiRNAs, the OSsRNA-3- *LOC_Os05g43650.1*-phasiRNA and OSsRNA-14- *LOC_Os01g37325.1*-phasiRNA pathways are required for the rice development. Transponsons and retrotransponsons that play important roles in plant gene and genome evolution are ubiquitous in plants [47]. We hypothesized that the transcripts of transponson and retrotransponson might also function as important sources of phasiRNA in plants. Further exploration of such phasiRNA biogenesis pathways could benefits the in-depth investigation of their biogenesis mechanism and the miRNA/sRNA directed regulatory networks in plants.

For those phasiRNAs generated from the transcripts of *LOC_Os01g57968.1*, *LOC_Os02g20200.1*, *LOC_Os02g55550.1*, *LOC_Os04g45834.2* and *LOC_Os09g14490.1*, none of their targets were identified. However, considering these phasiRNAs were detected only in seedling, it still cannot rule out the possibility that these phasiRNA biogenesis pathways might take place in rice seedling development. *LOC_Os04g45834.2* encodes a DUF584 domain containing protein. These protein family has been involved in leaf senescence in plant [48]. *LOC_Os09g14490.1* encodes a TIR-NBS type disease resistance protein, which has been identified in resistance to multiple viruses in plant [49–51]. *LOC_Os02g55550.1* encodes a F-box/LRR-repeat protein 14, which is involved in plant immune response [52]. These genes have been proved to play important roles in plants, however, their capability of producing secondary phasiRNAs suggest they might be involved in much more complex function than what we expected. Similarly, no targets of *LOC_Os01g57968.1*-derived phasiRNAs was identified, however, since these phasiRNAs only expressed in panicle tissue under normal condition, it might suggest the OSsRNA-1- *LOC_Os01g57968.1*-phasiRNA pathway might related to the rice panicle development. Thus, systematically investigation of the temporal and spatial expression specificity of phasiRNAs generated from the transcripts of protein-coding genes in our future work might gain insight into these phasiRNAs biogenesis requirement mechanism.

In this study, two cDNA sequences, *LOC_Os09g00999.1* and *LOC_Os09g01000.1*, which were able to produce plenty of Dicer-independent secondary siRNAs in most of tissues, have attracted our attention. We further employed the searching of phasiRNAs generated from *LOC_Os09g00999.1* and *LOC_Os09g01000.1* for target prediction and identification. The results indicated plenty of siRNA-target interaction pairs were discovered (data not shown). This might suggest a novel pattern of secondary siRNAs biogenesis pathways. Therefore, further investigation of Dicer-independent secondary siRNAs biogenesis pathways in plant might provide more strong evidence of this biogenesis pattern, and more meaningful information of the small RNA regulatory mechanism in plant.

## Conclusions

Here, we performed degradome-based screening of novel phasiRNA biogenesis pathways in rice. Five novel 21-nt phasiRNA biogenesis pathways and five novel 24-nt phasiRNA biogenesis pathways were also identified in addition to two known 21-nt phasiRNA biogenesis pathways. Further analysis on the targets of these novel phasiRNAs in 21-nt and 22-nt length revealed that eleven novel phasiRNAs mediated forty-one siRNA-target interactions during rice growth and development (Table 2, Additional file 1: Table S1, Additional file 2: Table S2 and Additional file 6: Figure S3). These results demonstrated the effectiveness of degradome-based screening in mining novel phasiRNA biogenesis pathways and substantially extend the information of phasiRNA biogenesis pathways in rice. We believed that, more novel phasiRNA biogenesis pathways might be identified if extend our approach to other plant species.

## Methods

### Data source

The *Oryza sativa* sRNA HTS datasets of seedling, root, shoot and panicle samples under normal (control) and stress conditions, the sRNA HTS datasets of wild type, *osdcl4* and *osdcl3* mutants and the degradome sequencing datasets were retrieved from GEO (Gene Expression Omnibus; http://www.ncbi.nlm.nih.gov/geo/). The bisulfite-seqand RNA-seq datasets of panicle and root were contributed by Zhao et al. [41]. All the HTS datasets employed for our study were listed in Additional file 5: Table S3.

The cDNAs, full-length genomic sequences of *Oryza sativa* were retrieved from PlantGDB (http://plantgdb.org/XGDB/phplib/). The promoter sequences of *Oryza sativa* were retrieved from PlantProm DB (http://linux1.softberry.com/). All the high-throughput sequencing data were pre-processed before use, the data of each library was normalized in RPM (reads per million) as described in our previous report [53].

### Identification of phasiRNA biogenesis pathways in *Oryza sativa*

The phasiRNA loci identification criteria were established based on the revised trans-acting siRNA (tasiRNA) biogenesis model as we reported previously [28]. The screening of PHAS loci in rice was followed by four steps: (1) cDNA/genome sequences-derived 21-nt phased duplexes were computational predicted by “phase processing”, each of these duplexes has a 2-nt overhang at 3′-end. (2) Each of these duplexes was separated into two increments and used for matching with small RNAs from small RNA high throughput sequencing datasets of Rice. A potential phasiRNA production region shall contain at least 5 tandem “processing” duplexes and each of these duplexes shall contains detectable phasiRNA from sense strand (plus siRNA) and/or antisense strand (minus siRNA). (3) Degradome HTS libraries which contributed by the works of Wu et al. [54], Li et al. [55] and Zhou et al. [56] were employed for systematically scanning the degradome-supported cleavage signatures on the screened possible phasiRNA production regions as we described in our previous work [28], and maintain the PHAS loci candidates with cleavage signatures which located in the phasiRNA production region. (4) The sRNAs bound to the PHAS loci were analyzed by using miRU algorithm [57], and the sRNA cleavage sites on those loci were further verified by using degradome sequencing libraries. The degradome-supported cleavage site of a sRNA trigger shall reside within 10 to 11-nt from the 5′ end of the binding site [58]. (5) The phasing score of phasiRNA production from each PHAS loci candidate should above 1.

### Calculation of phasing score

Phasing scores of phasiRNA regions were calculated based on the formula which contributed by Zheng et al. [23]: $\mathrm{Phasing}\ \mathrm{score}=1\mathrm{n}\Big[{\left(1+10\times \frac{\sum_{i=1}^5 pi}{1+\sum U}\right)}^{n-2}$, where N represents the number of phase register occupied by at least one unique 21-nt/24-nt small RNA within a five-phase register window, p represents the total number of reads for all 21-nt/24-nt small RNA falling into a given phase in a given window, U represents the total number of unique reads for all 21-nt/24-nt small RNA falling out of a given phase.

### Identitification of phasiRNA-target interaction based on degradome sequencing

The expressed novel phasiRNAs generated from 21-nt PHAS loci were predicted based on previously modified model of tasiRNA biogenesis in plant [28]. The predicted phasiRNAs were recruited for target prediction by using miRU with default parameters [57], and followed by degradome sequencing-based verification, as described previously [53, 59].

### Gene expression level analysis

The sequences of RNA-seq datasets were mapped to the reference cDNA sequences, and each gene expression level was calculated by the total RPM of mapped sequences.

### Identification of 24 nt phasiRNA target

In order to identify the potential 24-nt phasiRNA target sites in promoter sequences, BLAST analysis was performed for finding the location of the complementary sequence of 24-nt phasiRNA with no mismatch [60]. The promoters possessed phasiRNA binding sites were remained as potential target promoters. As each of the downloaded promoter sequence containing partial mRNA sequence, we identified the corresponding potential target genes by mapping the partial mRNA sequence to cDNA sequences. The DNA methylation status of potential target promoters were analyzed by utilizing the bisulfite-seq datasets of panicle and root of rice. The expression specificity of phasiRNA in different tissues should consistent with the occurring of increasing methylation of the target promoter.

The DNA methylation analysis of promoters were performed according to the method developed by Zhao et al. [41]. The sequences of bisulfite sequencing libraries were mapped to the potential promoter sequences, and the uniquely mapped sequences were used for further DNA methylation level analysis. The DNA methylation level of each cytosine was obtained by calculation of the total coverage of individual cytosines in RPM.

## Supplementary Information

**Additional file 1: Table S1.** Identification of 21-nt PHAS loci in Rice

**Additional file 2: Table S2.** Identification of 24-nt PHAS loci in Rice

**Additional file 3: Figure S1.** 21-nt phasiRNAs generated from the novel PHAS loci in wild-type and DCL4 mutant*.* 21-nt phasiRNAs generated from transcripts of *LOC_Os01g57968.1* (A) and LOC_Os05g43650.1 (D) in wild-type and *osdcl4*-1 seedling, *LOC_Os02g18750.1* (B), *LOC_Os04g25740.1* (C) and LOC_Os06g30680.1(E) in wild-type and *osdcl4*-1 mutant panicle, respectively. The black arrows indicate the sRNA trigger cleavage sites, the x-axis represent the phasiRNA position mapped within the PHAS loci, the y-axis represent the read abundance (in RMP, reads per million) of the small RNAs mapped to the sense and antisense strands of PHAS loci.

**Additional file 4: Figure S2.** 24-nt phasiRNAs generated from the novel PHAS lociin wild-type and DCL3 mutant*.* 24-nt phasiRNAs generated from transcripts of *LOC_Os01g37325.1* (A), *LOC_Os02g20200.1* (B), *LOC_Os02g55550.1* (C), *LOC_Os04g45834.2* (D) and *LOC_Os09g14490.1* (E) in wild-type and *osdcl3*–1 mutant seedling, respectively. The black arrows indicate the sRNA trigger cleavage sites, the x-axis represent the phasiRNA position mapped within the PHAS loci, the y-axis represent the read abundance (in RMP, reads per million) of the small RNAs mapped to the sense and antisense strands of PHAS loci.

**Additional file 5: Table S3.** Datasets utilized for our study.

**Additional file 6: Figure S3.** Degradome sequencing-based validation of the phasiRNA-target interactions.Four libraries of degradome sequencing data libraries (GSM434596, GSM455938, GSM455939 and GSM476257) were recruited for T-plot profiling**.** The IDs of the target transcripts and the corresponding phasiRNAs generated from the transcripts of *LOC_Os02g18750.1*, *LOC_Os05g43650.1*, *LOC_Os06g30680.1* and *LOC_Os12g42380.1* are listed on the top. The *y* axis measure the normalized reads (in RMP, reads per million) of the degradome signals, and the *x* axis represent the position of the cleavage signals on the target transcripts. The binding sites of the phasiRNA on their target transcripts were denoted by gray horizontal lines, and the dominant cleavage signals were marked by black arrows.

## Data Availability

The sRNA HTS datasets of *Oryza sativa* seedling, root, shoot and panicle samples under normal (control) and stress conditions, which employed for searching PHAS loci are available at the NCBI GEO DataSets under the accession numbers GSM455965 and GSE32973. The sRNA HTS datasets used for evaluating the phasiRNA production from PHAS loci in wild type, *osdcl4* and *osdcl3* mutants are available at the NCBI GEO DataSets under the accession number GSM562942, GSM562943, GSM562944, GSM562945,GSM520638 and GSM520640. The degradome sequencing datasets utilized for PHAS loci identification and tasiRNAs’ targets verification are available at NCBI GEO DataSets under the accession number GSM434596, GSM455938, GSM455939 and GSM476257. The bisulfite-seq and RNA-seq datasets used for identification of 24-nt phasiRNA mediated RdDM in rice are available at NCBI GEO DataSets under the accession number GSM4232038, GSM4232039, GSM4230036, GSM4230037, GSM4230038 and GSM4230039. The cDNAs, genome sequences of rice are available at PlantGDB (http://plantgdb.org/XGDB/phplib/) and the promoter sequences of rice are available at PlantProm DB (http://linux1.softberry.com/).

## Abbreviations

phasiRNAs: Phased small interfering RNAs
siRNAs: Small interfering RNAs
miRNAs: microRNAs
AGO: ARGONATE
RISC: RNA-induced silencing complex
dsRNA: Double-stranded RNA
RDR6: RNA-dependent RNA polymerase 6
SGS3: Suppressor of Gene Silencing 3
DCL: Dicer-like
phasiRNAs: Phased small interfering RNAs
PHAS loci: PhasiRNA production precursors
sRNA: Small RNA
tasiRNA: Trans-acting siRNA
RdDM: RNA directed DNA methylation

## References

[1] Hohn T, Vazquez F (2011). RNA silencing pathways of plants: silencing and its suppression by plant DNA viruses. Biochimica et biophysica acta.

[2] Mermigka G, Verret F, Kalantidis K (2016). RNA silencing movement in plants. Journal of integrative plant biology.

[3] Yu D, Lu J, Shao W, Ma X, Xie T, Ito H, Wang T, Xu M, Wang H, Meng Y. MepmiRDB: a medicinal plant microRNA database. Database (Oxford). 2019;2019:baz070. 10.1093/database/baz070 PMC6589547 31231773

[4] Singh A, Gautam V, Singh S, Sarkar Das S, Verma S, Mishra V, Mukherjee S, Sarkar AK (2018). Plant small RNAs: advancement in the understanding of biogenesis and role in plant development. Planta.

[5] Bian H, Xie Y, Guo F, Han N, Ma S, Zeng Z, Wang J, Yang Y, Zhu M (2012). Distinctive expression patterns and roles of the miRNA393/TIR1 homolog module in regulating flag leaf inclination and primary and crown root growth in rice (Oryza sativa). The New phytologist.

[6] Zhu C, Ding Y, Liu H (2011). MiR398 and plant stress responses. Physiologia plantarum.

[7] Guleria P, Mahajan M, Bhardwaj J, Yadav SK (2011). Plant small RNAs: biogenesis, mode of action and their roles in abiotic stresses. Genomics, proteomics & bioinformatics.

[8] Yu Y, Zhang Y, Chen X, Chen Y, Plant Noncoding RNA (2019). Hidden Players in Development and Stress Responses. Annual review of cell and developmental biology.

[9] Fei Q, Xia R, Meyers BC (2013). Phased, secondary, small interfering RNAs in posttranscriptional regulatory networks. The Plant cell.

[10] Li S, Liu J, Liu Z, Li X, Wu F, He Y (2014). HEAT-INDUCED TAS1 TARGET1 Mediates Thermotolerance via HEAT STRESS TRANSCRIPTION FACTOR A1a-Directed Pathways in Arabidopsis. The Plant cell.

[11] Matsui A, Mizunashi K, Tanaka M, Kaminuma E, Nguyen AH, Nakajima M, Kim JM, Nguyen DV, Toyoda T (2014). Seki M: tasiRNA-ARF pathway moderates floral architecture in Arabidopsis plants subjected to drought stress. BioMed research international.

[12] Axtell MJ, Jan C, Rajagopalan R, Bartel DP (2006). A two-hit trigger for siRNA biogenesis in plants. Cell.

[13] D'Ario M, Griffiths-Jones S, Kim M, Small RNA (2017). Big Impact on Plant Development. Trends in plant science.

[14] Xia R, Xu J, Meyers BC (2017). The Emergence, Evolution, and Diversification of the miR390-TAS3-ARF Pathway in Land Plants. The Plant cell.

[15] Luo QJ, Mittal A, Jia F, Rock CD (2012). An autoregulatory feedback loop involving PAP1 and TAS4 in response to sugars in Arabidopsis. Plant molecular biology.

[16] Law JA, Jacobsen SE (2010). Establishing, maintaining and modifying DNA methylation patterns in plants and animals. Nature reviews Genetics.

[17] Liu Q, Ding C, Chu Y, Zhang W, Guo G, Chen J (2017). Su X: Pln24NT: a web resource for plant 24-nt siRNA producing loci. Bioinformatics.

[18] Li C, Xu H, Fu FF, Russell SD, Sundaresan V, Gent JI (2020). Genome-wide redistribution of 24-nt siRNAs in rice gametes. Genome research.

[19] Fan Y, Yang J, Mathioni SM, Yu J, Shen J, Yang X, Wang L, Zhang Q, Cai Z, Xu C (2016). PMS1T, producing phased small-interfering RNAs, regulates photoperiod-sensitive male sterility in rice. Proc Natl Acad Sci U S A.

[20] Song X, Li P, Zhai J, Zhou M, Ma L, Liu B, Jeong DH, Nakano M, Cao S, Liu C (2012). Roles of DCL4 and DCL3b in rice phased small RNA biogenesis. The Plant J.

[21] Ma W, Chen C, Liu Y, Zeng M, Meyers BC, Li J, Xia R (2018). Coupling of microRNA-directed phased small interfering RNA generation from long noncoding genes with alternative splicing and alternative polyadenylation in small RNA-mediated gene silencing. New Phytol.

[22] Liu Y, Wang Y, Zhu QH, Fan L. Identification of phasiRNAs in wild rice (Oryza rufipogon). Plant Signal Behav. 2013;8(8). 10.4161/psb.25079 PMC4005798 23733069

[23] Zheng Y, Wang Y, Wu J, Ding B, Fei Z (2015). A dynamic evolutionary and functional landscape of plant phased small interfering RNAs. BMC biology.

[24] Komiya R (2017). Biogenesis of diverse plant phasiRNAs involves an miRNA-trigger and Dicer-processing. Journal of plant research.

[25] Yu L, Guo R, Jiang Y, Ye X, Yang Z, Meng Y, Shao C (2019). Identification of novel phasiRNAs loci on long non-coding RNAs in Arabidopsis thaliana. Genomics.

[26] Liu B, Chen Z, Song X, Liu C, Cui X, Zhao X, Fang J, Xu W, Zhang H, Wang X (2007). Oryza sativa dicer-like4 reveals a key role for small interfering RNA silencing in plant development. The Plant cell.

[27] Huang J, Wang R, Dai X, Feng J, Zhang H, Zhao PX (2019). A microRNA biogenesis-like pathway for producing phased small interfering RNA from a long non-coding RNA in rice. Journal of experimental botany.

[28] Yu L, Meng Y, Shao C, Kahrizi D (2015). Are ta-siRNAs only originated from the cleavage site of miRNA on its target RNAs and phased in 21-nt increments?. Gene.

[29] Boonburapong B, Buaboocha T (2007). Genome-wide identification and analyses of the rice calmodulin and related potential calcium sensor proteins. BMC Plant Biol.

[30] Rouhier N, Couturier J, Jacquot JP (2006). Genome-wide analysis of plant glutaredoxin systems. J Exp Bot.

[31] Ma H, Zhao J (2010). Genome-wide identification, classification, and expression analysis of the arabinogalactan protein gene family in rice (Oryza sativa L.). J Exp Bot.

[32] Li X, Shahid MQ, Xia J, Lu Z, Fang N, Wang L, Wu J, Chen Z, Liu X (2017). Analysis of small RNAs revealed differential expressions during pollen and embryo sac development in autotetraploid rice. BMC Genomics.

[33] Dong S, Zhang J, Sun D, Liu H, Yang Q, Wang H, Chen Z, Wang J (2018). Identification of Magnaporthe oryzae-elicited rice novel miRNAs and their targets by miRNA and degradome sequencing. European Journal of Plant Pathology.

[34] Ding Y, Qu A, Gong S, Huang S, Lv B, Zhu C (2013). Molecular identification and analysis of Cd-responsive microRNAs in rice. J Agric Food Chem.

[35] Vranova E, Coman D, Gruissem W (2012). Structure and dynamics of the isoprenoid pathway network. Molecular plant.

[36] Zhou B, Mural RV, Chen X, Oates ME, Connor RA, Martin GB, Gough J, Zeng L (2017). A Subset of Ubiquitin-Conjugating Enzymes Is Essential for Plant Immunity. Plant Physiol.

[37] Shaar-Moshe L, Hubner S, Peleg Z (2015). Identification of conserved drought-adaptive genes using a cross-species meta-analysis approach. BMC Plant Biol.

[38] Campo S, Baldrich P, Messeguer J, Lalanne E, Coca M, San Segundo B (2014). Overexpression of a Calcium-Dependent Protein Kinase Confers Salt and Drought Tolerance in Rice by Preventing Membrane Lipid Peroxidation. Plant Physiol.

[39] Zeng F, Wu X, Qiu B, Wu F, Jiang L, Zhang G (2014). Physiological and proteomic alterations in rice (Oryza sativa L.) seedlings under hexavalent chromium stress. Planta.

[40] Zhu H, Kranz RG (2012). A nitrogen-regulated glutamine amidotransferase (GAT1_2.1) represses shoot branching in Arabidopsis. Plant physiology.

[41] Zhao L, Xie L, Zhang Q, Ouyang W, Deng L, Guan P, Ma M, Li Y, Zhang Y, Xiao Q (2020). Integrative analysis of reference epigenomes in 20 rice varieties. Nature communications.

[42] Liu W, Xu ZH, Luo D, Xue HW (2003). Roles of OsCKI1, a rice casein kinase I, in root development and plant hormone sensitivity. Plant J.

[43] Johnson C, Kasprzewska A, Tennessen K, Fernandes J, Nan GL, Walbot V, Sundaresan V, Vance V, Bowman LH (2009). Clusters and superclusters of phased small RNAs in the developing inflorescence of rice. Genome Res.

[44] Lu C, Chen J, Zhang Y, Hu Q, Su W, Kuang H (2012). Miniature inverted-repeat transposable elements (MITEs) have been accumulated through amplification bursts and play important roles in gene expression and species diversity in Oryza sativa. Mol Biol Evol.

[45] Creasey KM, Zhai J, Borges F, Van Ex F, Regulski M, Meyers BC, Martienssen RA (2014). miRNAs trigger widespread epigenetically activated siRNAs from transposons in Arabidopsis. Nature.

[46] Slotkin RK, Vaughn M, Borges F, Tanurdzic M, Becker JD, Feijo JA, Martienssen RA (2009). Epigenetic reprogramming and small RNA silencing of transposable elements in pollen. Cell.

[47] Lisch D (2013). How important are transposons for plant evolution?. Nat Rev Genet.

[48] Zheng X, Jehanzeb M, Habiba, Zhang Y, Li L, Miao Y. Characterization of S40-like proteins and their roles in response to environmental cues and leaf senescence in rice. BMC Plant Biol. 2019;19(1):174. 10.1186/s12870-019-1767-1 PMC6498481 31046677

[49] Xun H, Yang X, He H, Wang M, Guo P, Wang Y, Pang J, Dong Y, Feng X, Wang S (2019). Over-expression of GmKR3, a TIR-NBS-LRR type R gene, confers resistance to multiple viruses in soybean. Plant Mol Biol.

[50] Zhou X, Liu J, Bao S, Yang Y, Zhuang Y. Molecular Cloning and Characterization of a Wild Eggplant Solanum aculeatissimum NBS-LRR Gene, Involved in Plant Resistance to Meloidogyne incognita. Int J Mol Sci. 2018;19(2):583. 10.3390/ijms19020583 PMC5855805 29462897

[51] Fan JJ, Wang P, Xu X, Liu K, Ruan YY, Zhu YS, Cui ZH, Zhang LJ (2015). Characterization of a TIR-NBS-LRR gene associated with downy mildew resistance in grape. Genetics and molecular research : GMR.

[52] Matsushima N, Miyashita H (2012). Leucine-Rich Repeat (LRR) Domains Containing Intervening Motifs in Plants. Biomolecules.

[53] Yu L, Shao C, Ye X, Meng Y, Zhou Y, Chen M (2016). miRNA Digger: a comprehensive pipeline for genome-wide novel miRNA mining. Scientific reports.

[54] Wu L, Zhang Q, Zhou H, Ni F, Wu X, Qi Y (2009). Rice MicroRNA effector complexes and targets. The Plant cell.

[55] Li YF, Zheng Y, Addo-Quaye C, Zhang L, Saini A, Jagadeeswaran G, Axtell MJ, Zhang W, Sunkar R (2010). Transcriptome-wide identification of microRNA targets in rice. The Plant journal : for cell and molecular biology.

[56] Zhou M, Gu L, Li P, Song X, Wei L, Chen Z (2010). Cao X:Degradome sequencing reveals endogenous small RNA targets in rice (Oryza sativa L. ssp. indica). FrontBiol.

[57] Zhang Y. miRU: an automated plant miRNA target prediction server. Nucleic acids research. 2005;33(Web Server issue):W701–704. 10.1093/nar/gki383 PMC1160144 15980567

[58] Addo-Quaye C, Eshoo TW, Bartel DP, Axtell MJ (2008). Endogenous siRNA and miRNA targets identified by sequencing of the Arabidopsis degradome. Curr Biol.

[59] German MA, Luo S, Schroth G, Meyers BC, Green PJ (2009). Construction of Parallel Analysis of RNA Ends (PARE) libraries for the study of cleaved miRNA targets and the RNA degradome. Nature protocols.

[60] Baev V, Naydenov M, Apostolova E, Ivanova D, Doncheva S, Minkov I, Yahubyan G (2010). Identification of RNA-dependent DNA-methylation regulated promoters in Arabidopsis. Plant physiology and biochemistry : PPB.

