# Supplementary figures and images for: Degradome sequencing-based identification of phasiRNAs biogenesis pathways in Oryza sativa
Source: BMC Genomics. 2021 Jan 30;22:93. doi: 10.1186/s12864-021-07406-7 (PMC7847607; doi:10.1186/s12864-021-07406-7)

A

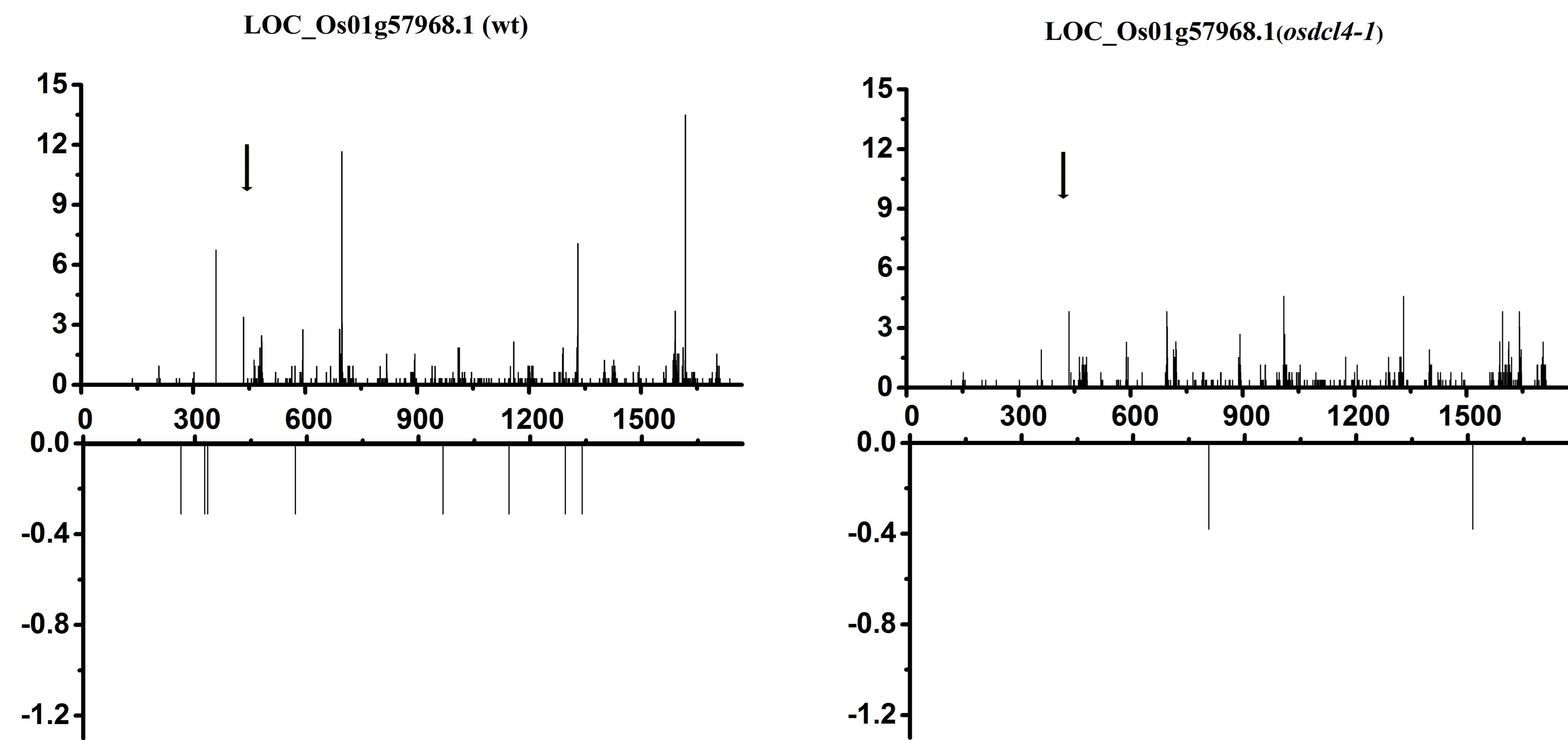

B

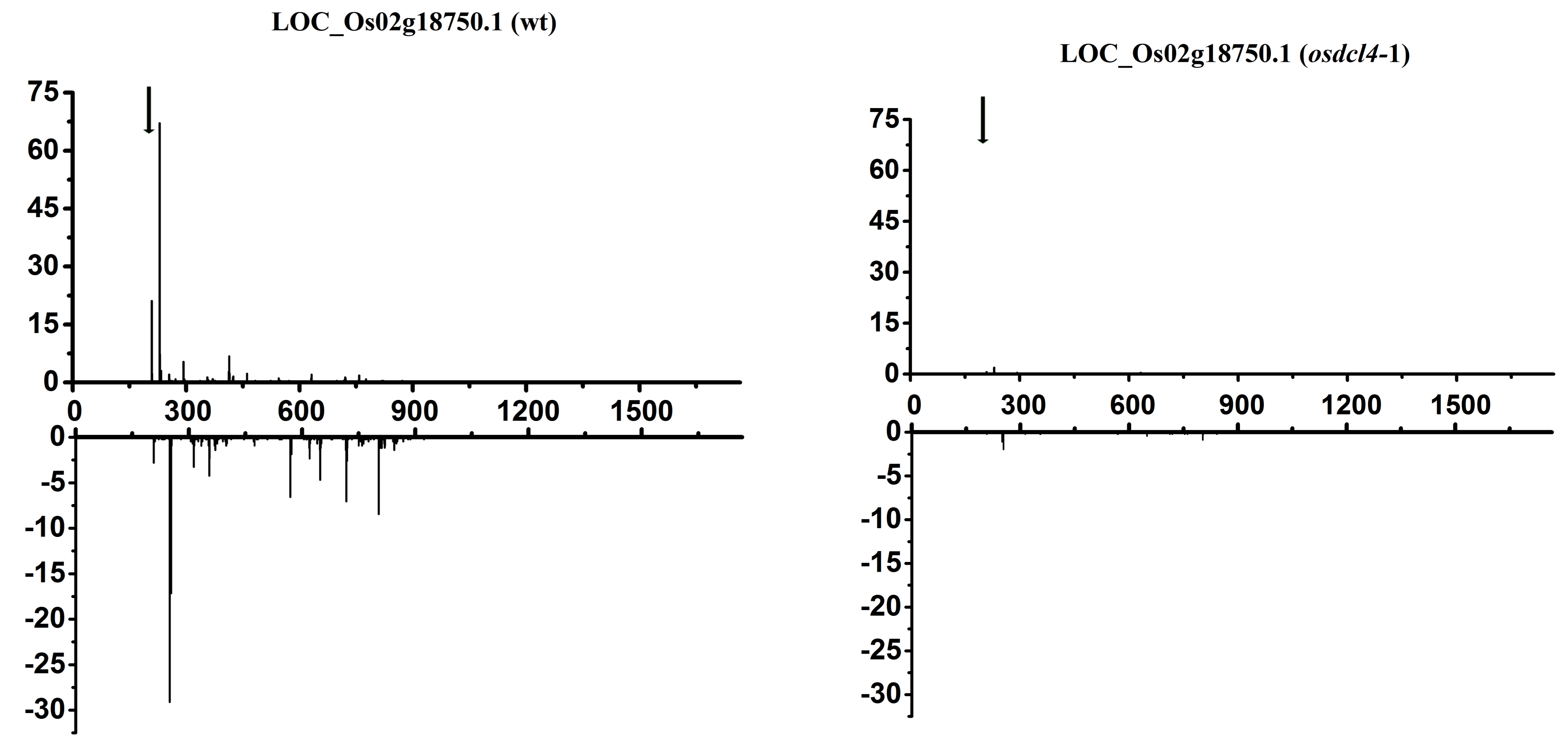

C

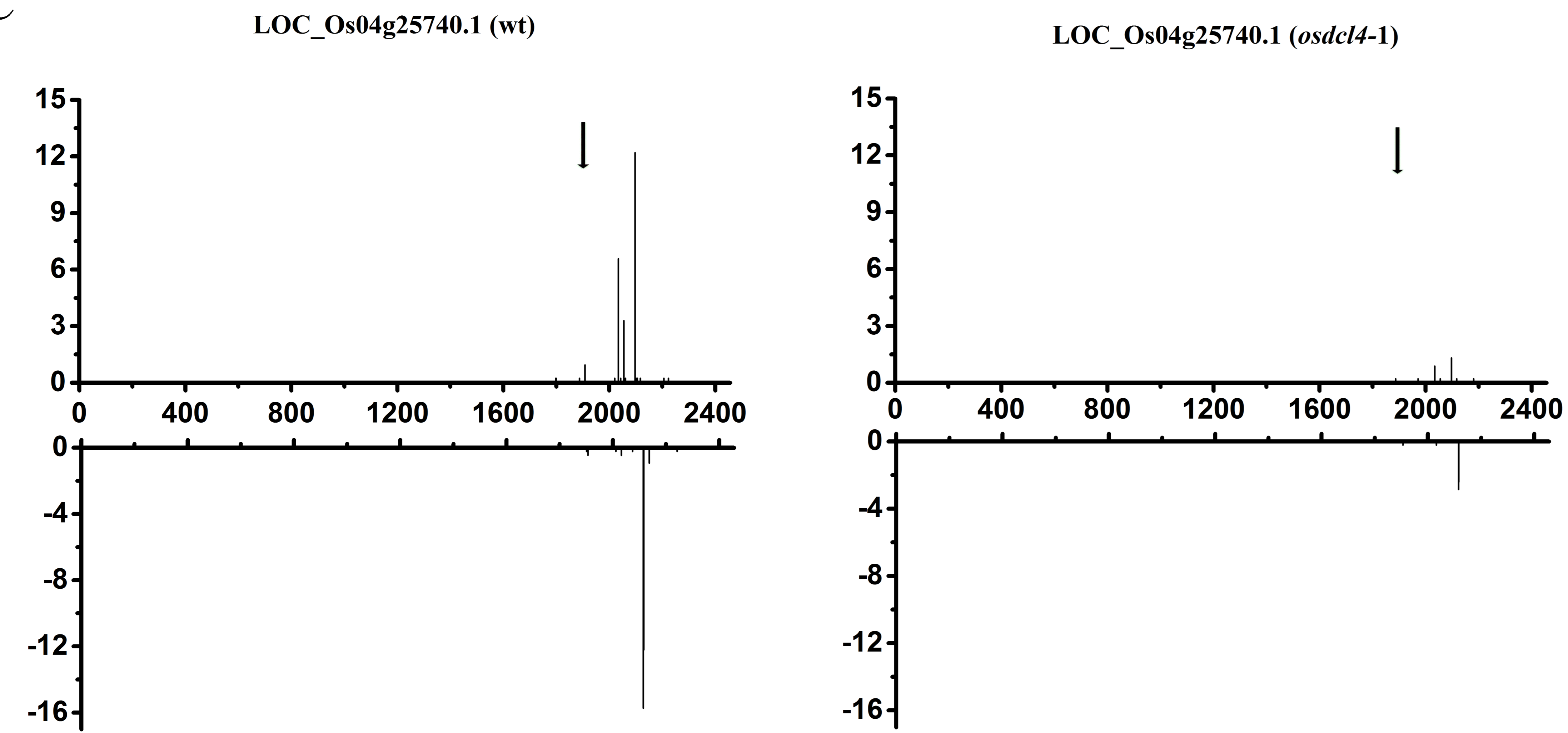

D

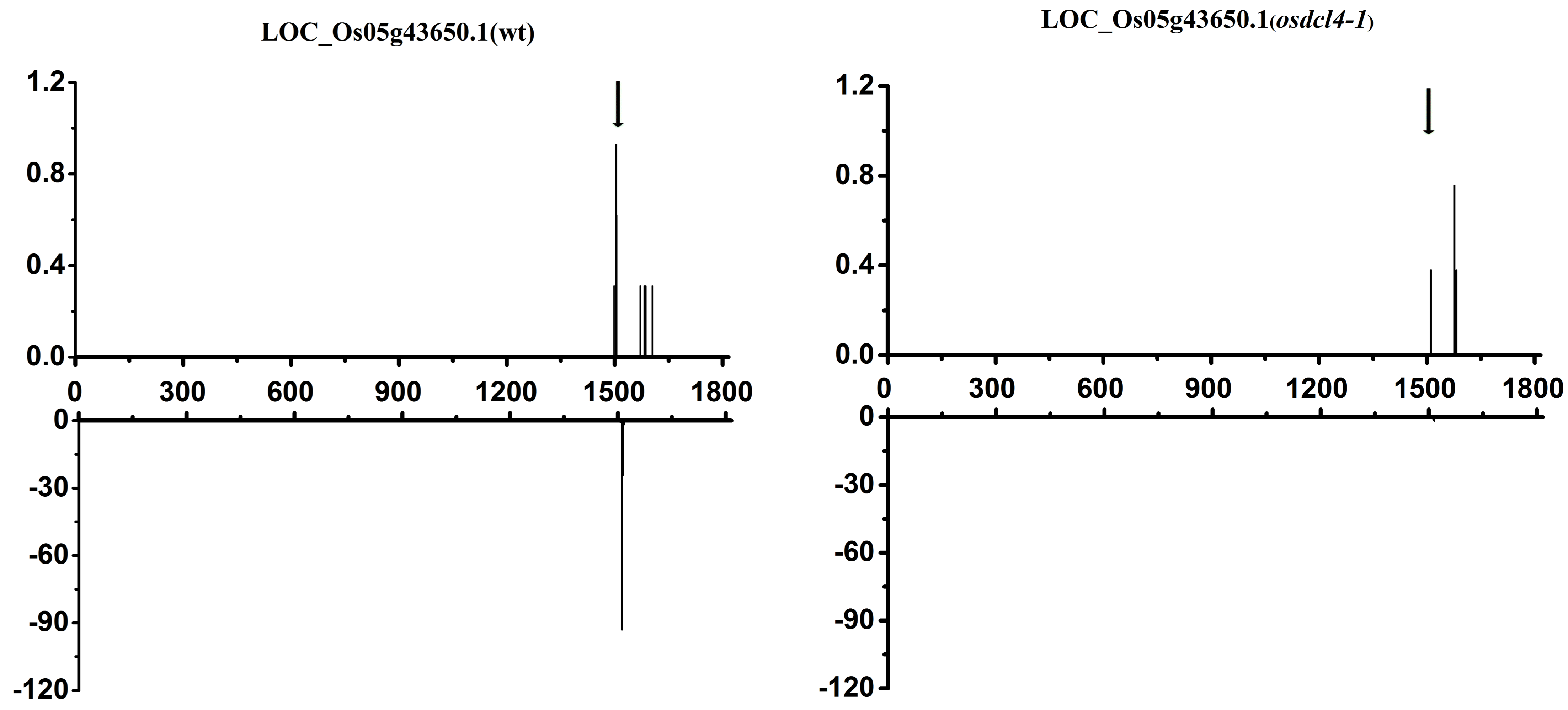

E

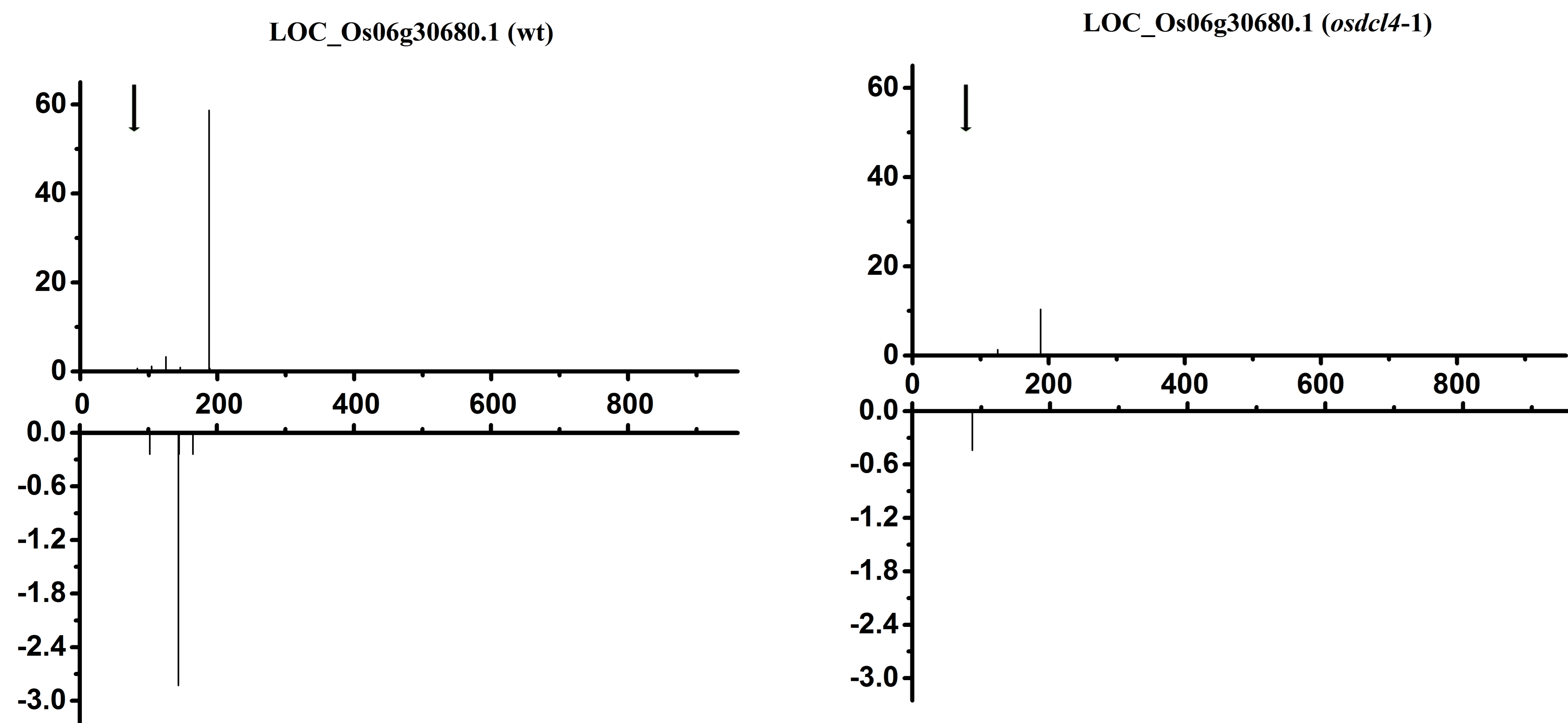

Supplement: Supplementary file 3 — Additional file 3: Figure S1. 21-nt phasiRNAs generated from the novel PHAS loci in wild-type and DCL4 mutant. 21-nt phasiRNAs generated from transcripts of LOC_Os01g57968.1 (A) and LOC_Os05g43650.1 (D) in wild-type and osdcl4-1 seedling, LOC_Os02g18750.1 (B), LOC_Os04g25740.1 (C) and LOC_Os06g30680.1(E) in wild-type and osdcl4-1 mutant panicle, respectively. The black arrows indicate the sRNA trigger cleavage sites, the x-axis represent the phasiRNA position mapped within the PHAS loci, the y-axis represent the read abundance (in RMP, reads per million) of the small RNAs mapped to the sense and antisense strands of PHAS loci. [file 12864_2021_7406_MOESM3_ESM.pdf]

A

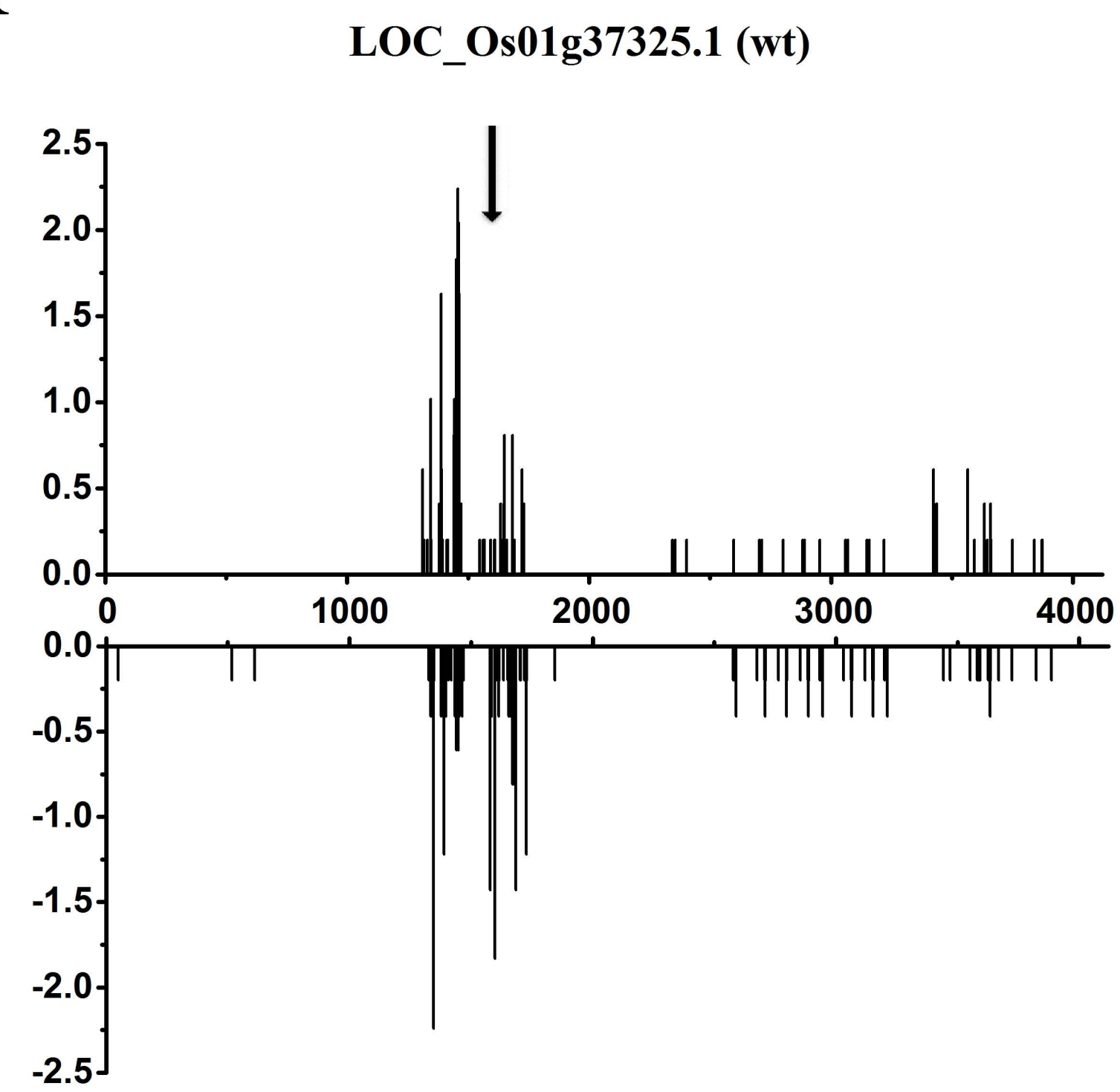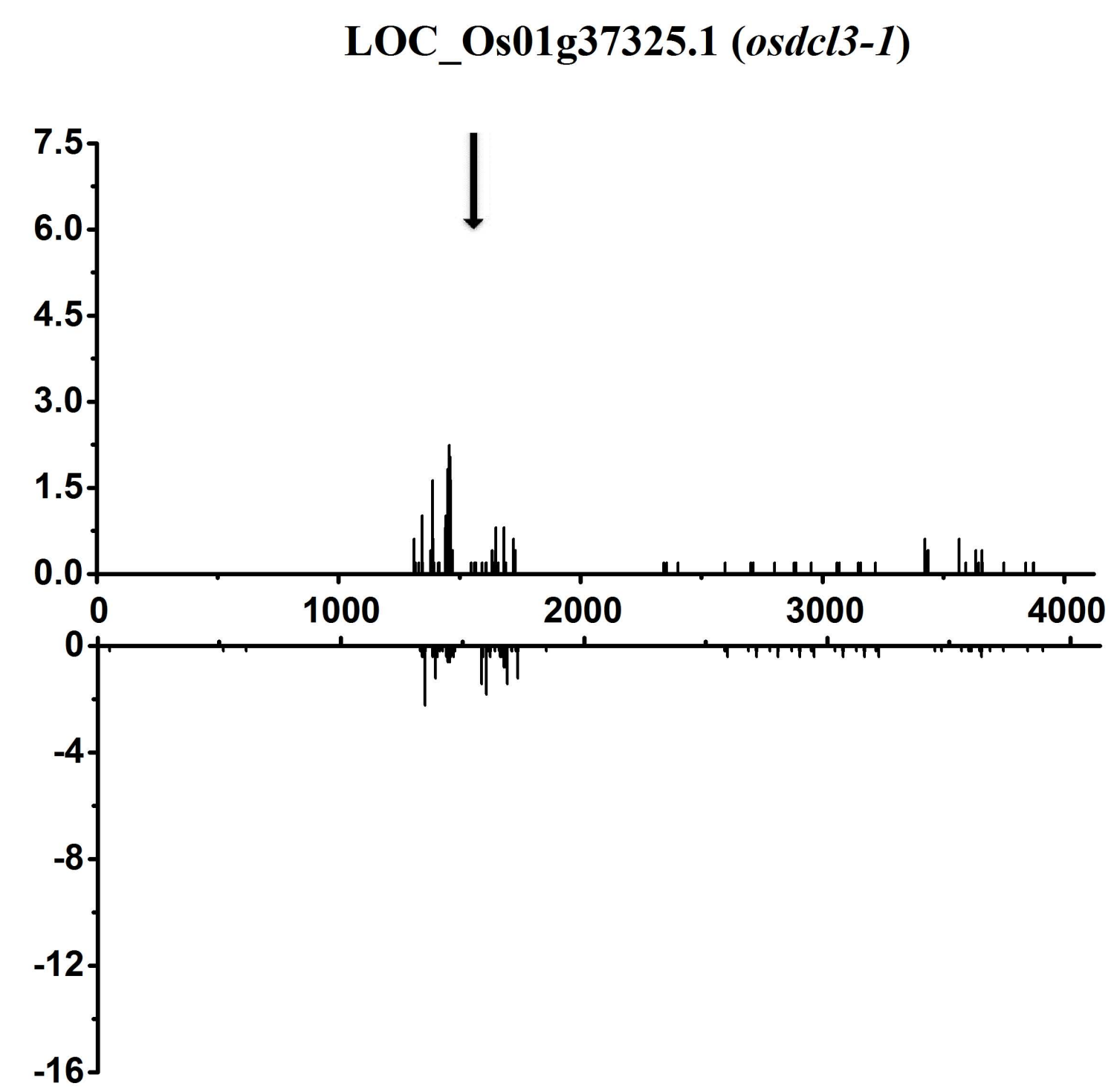

B

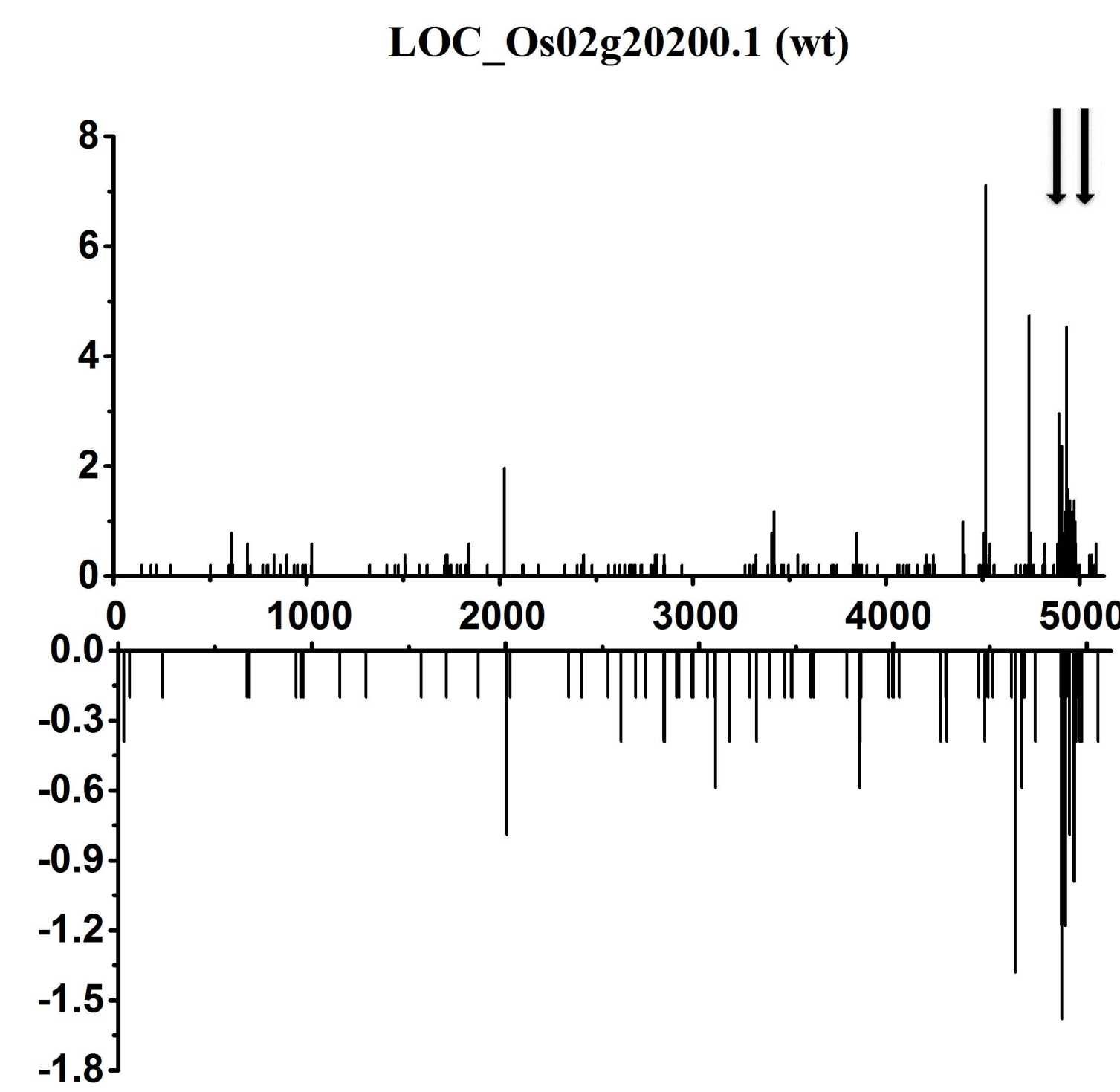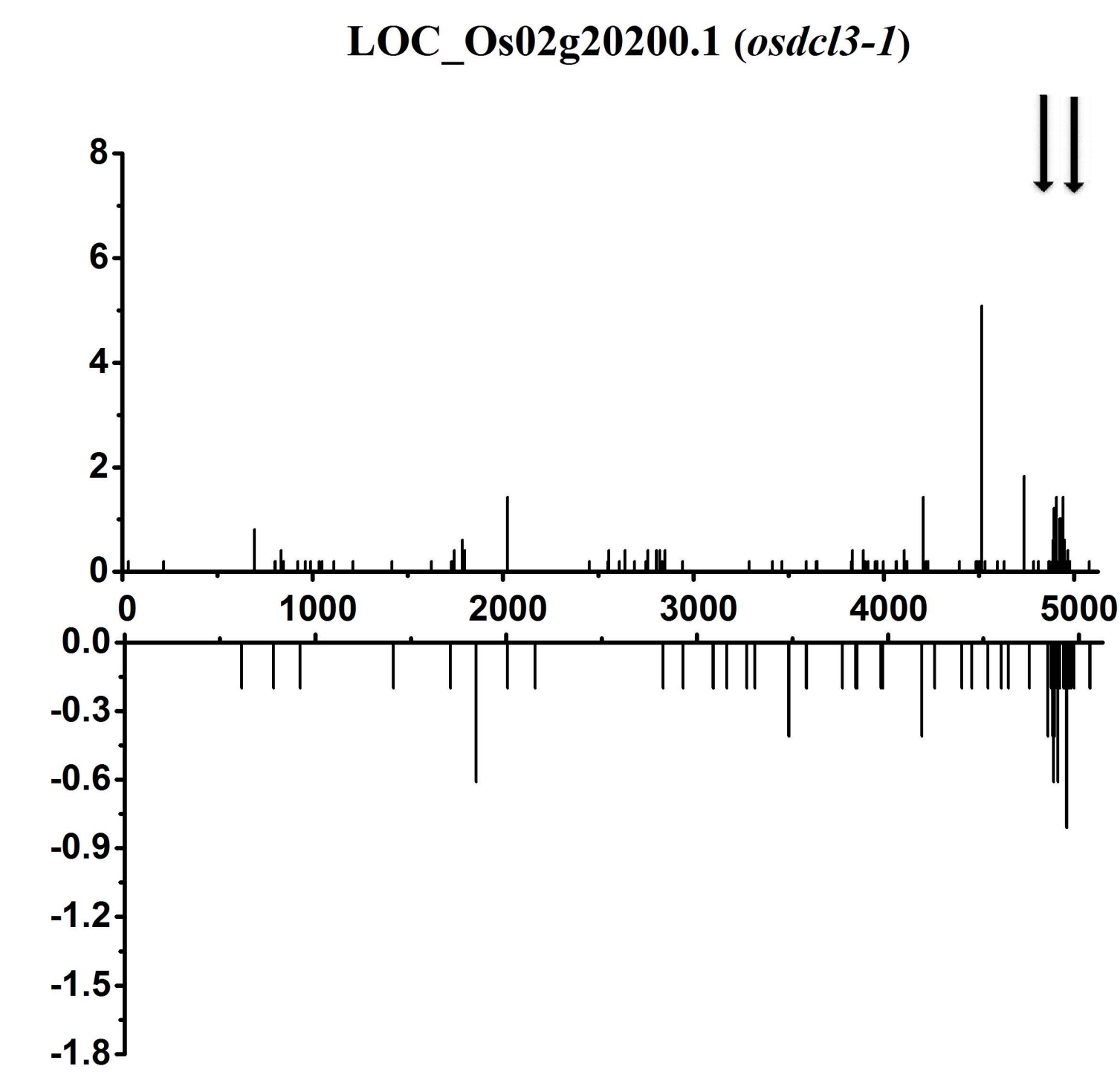

C

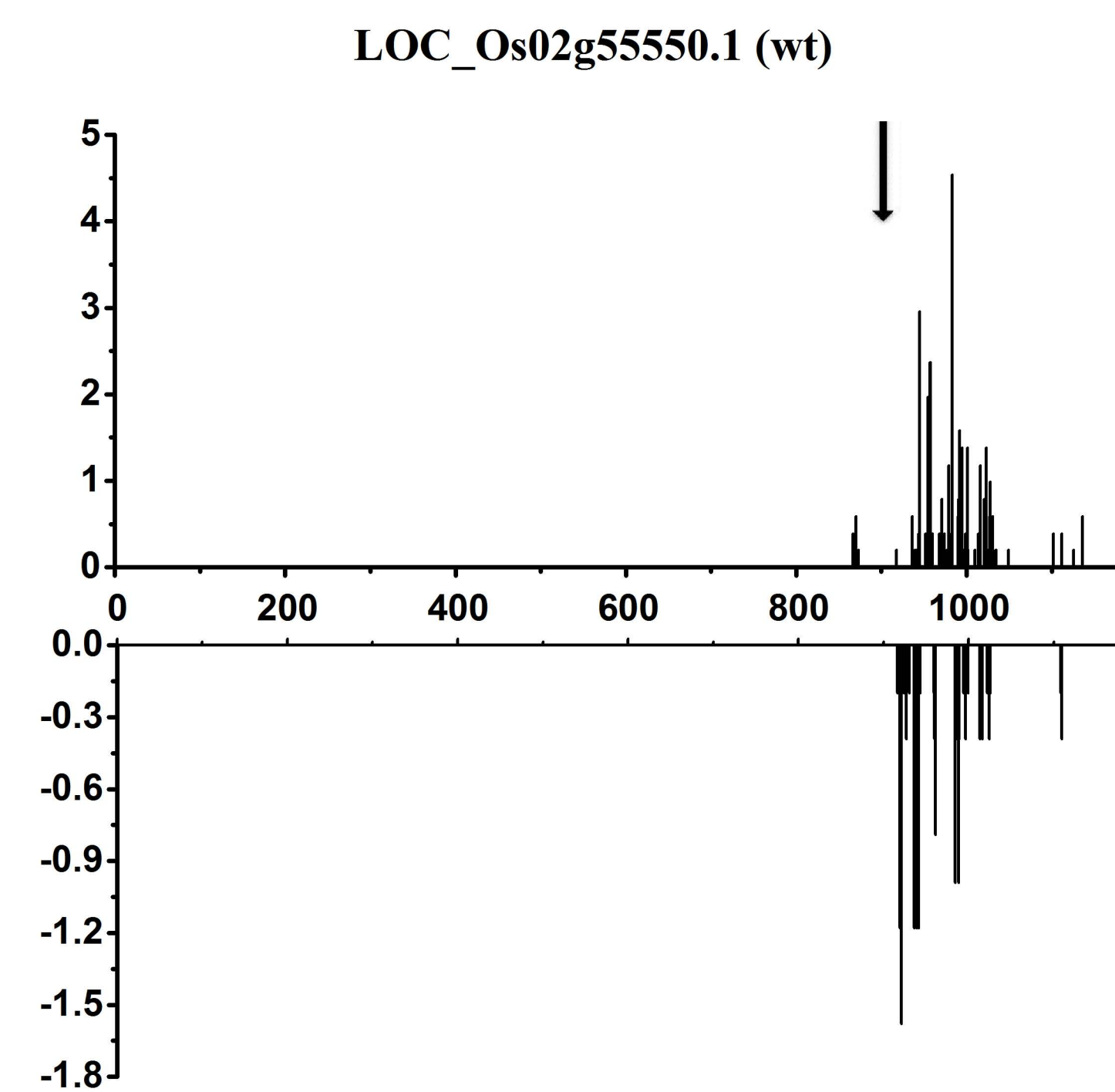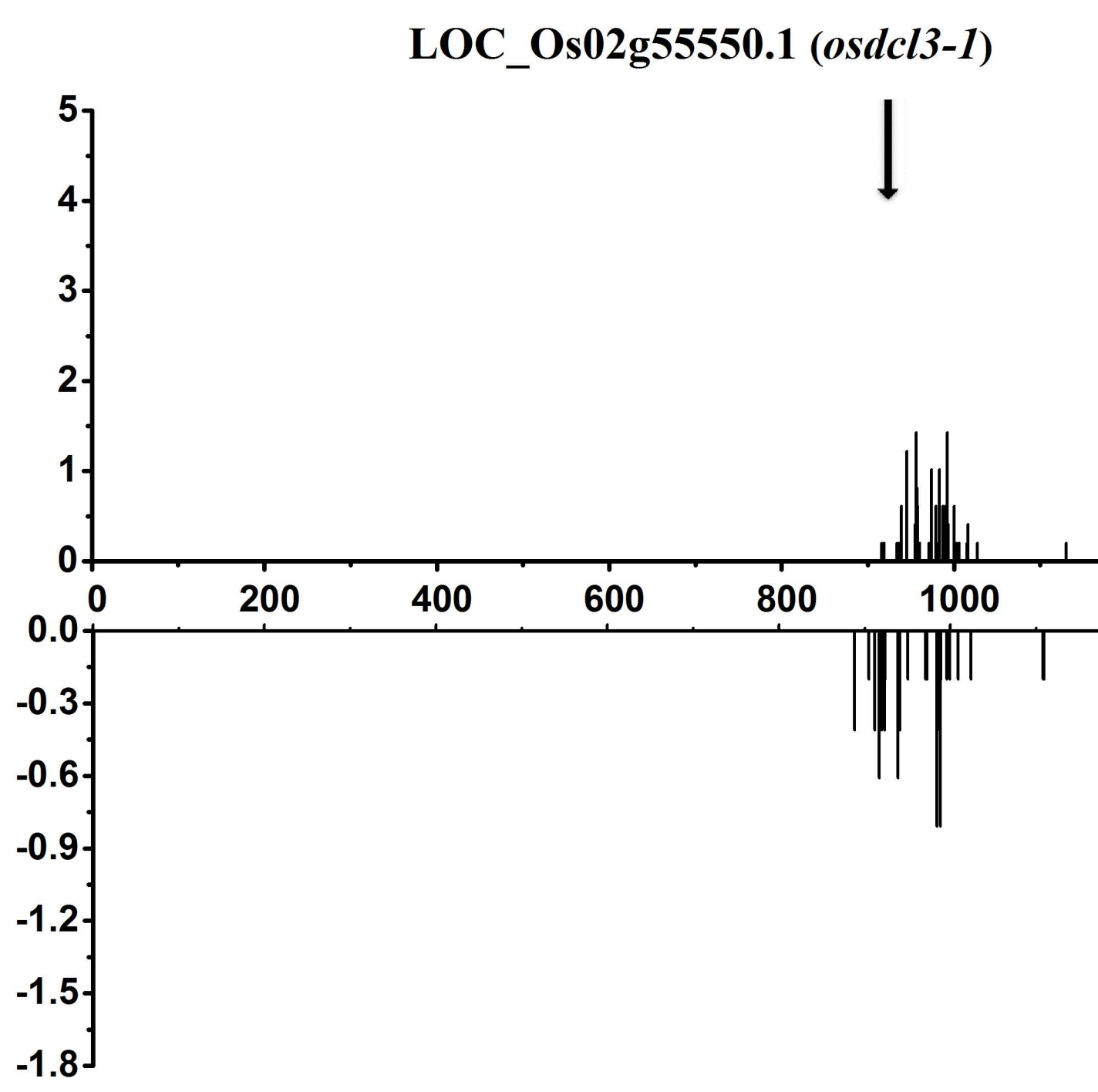

D

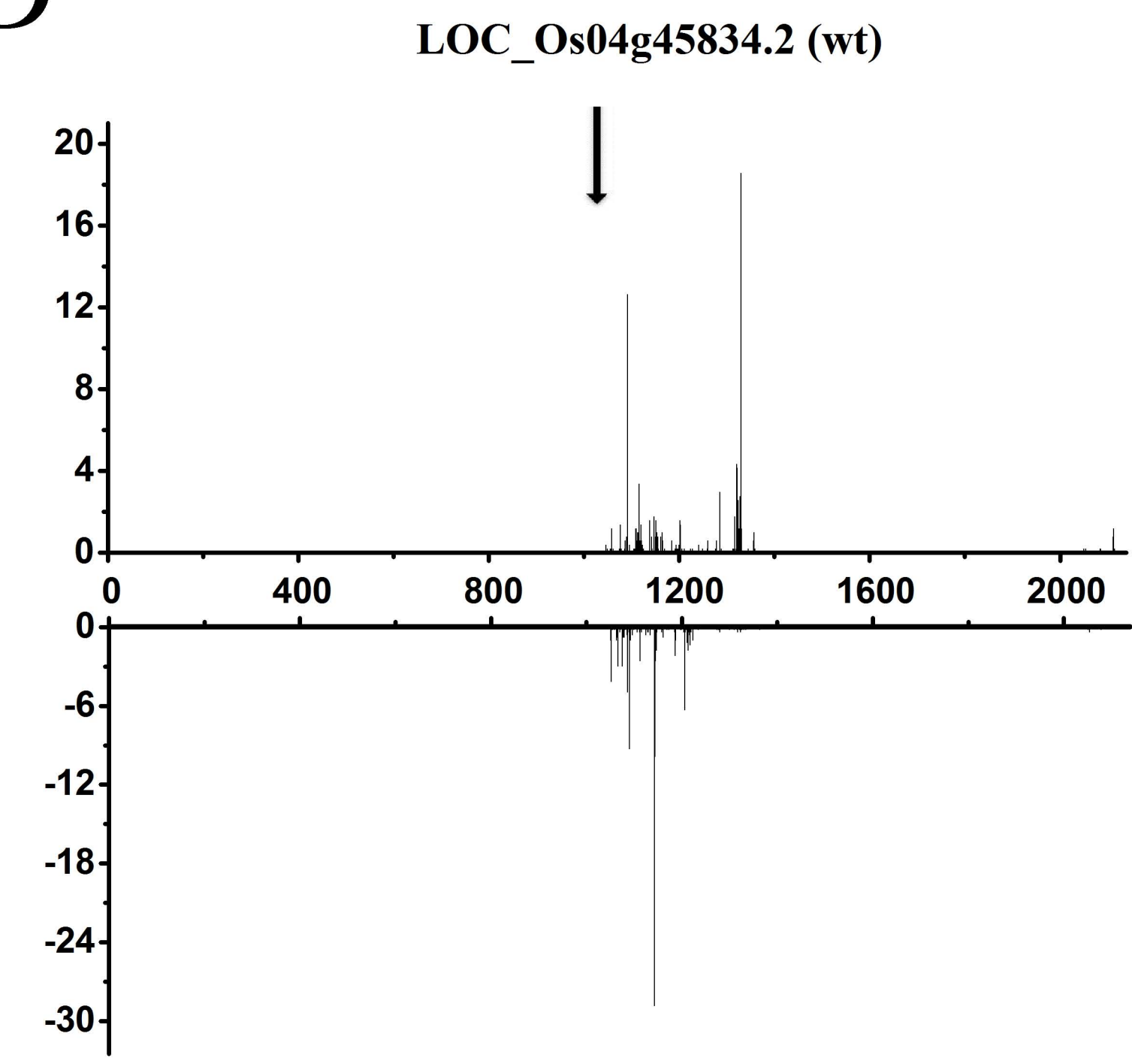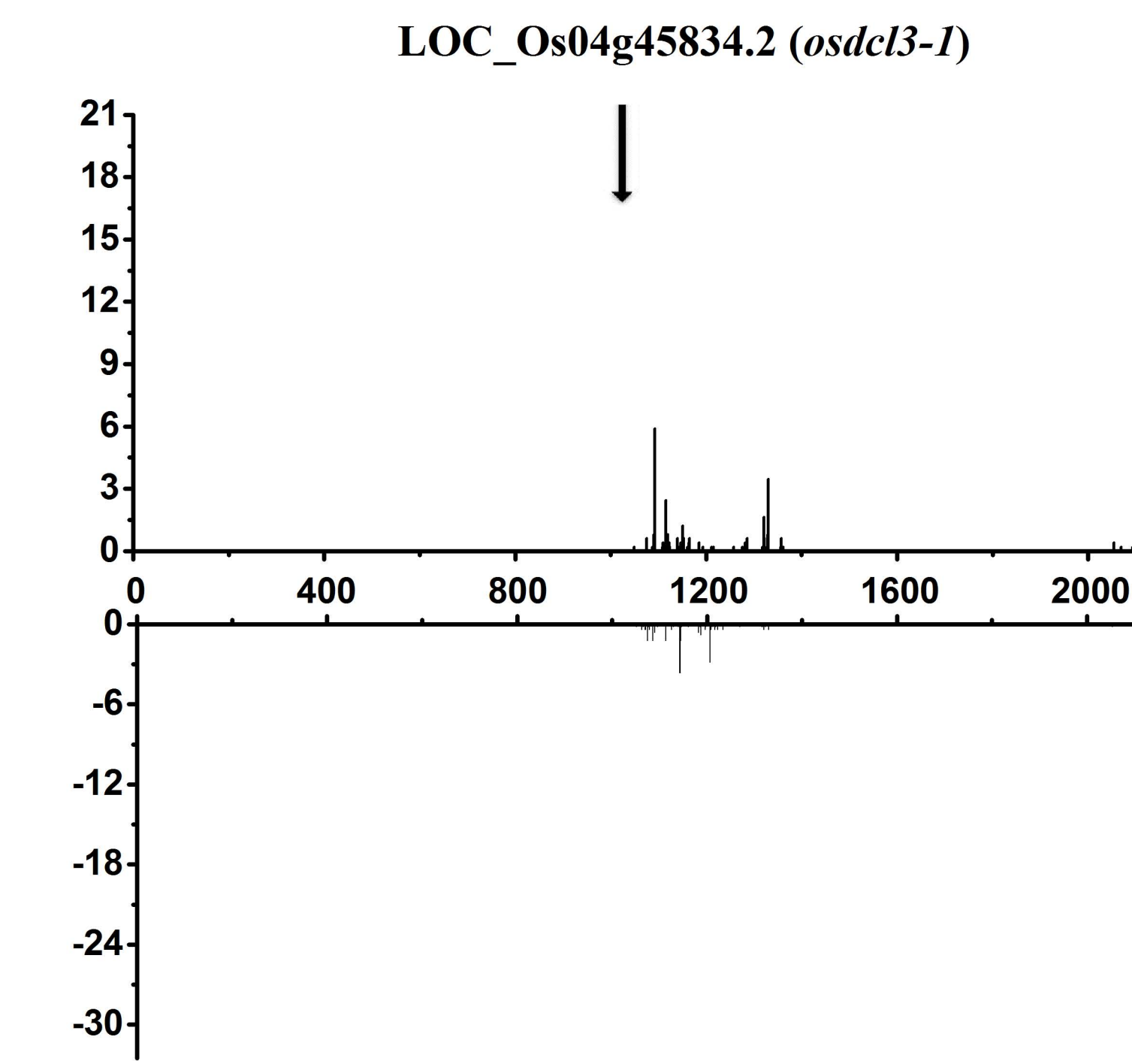

E

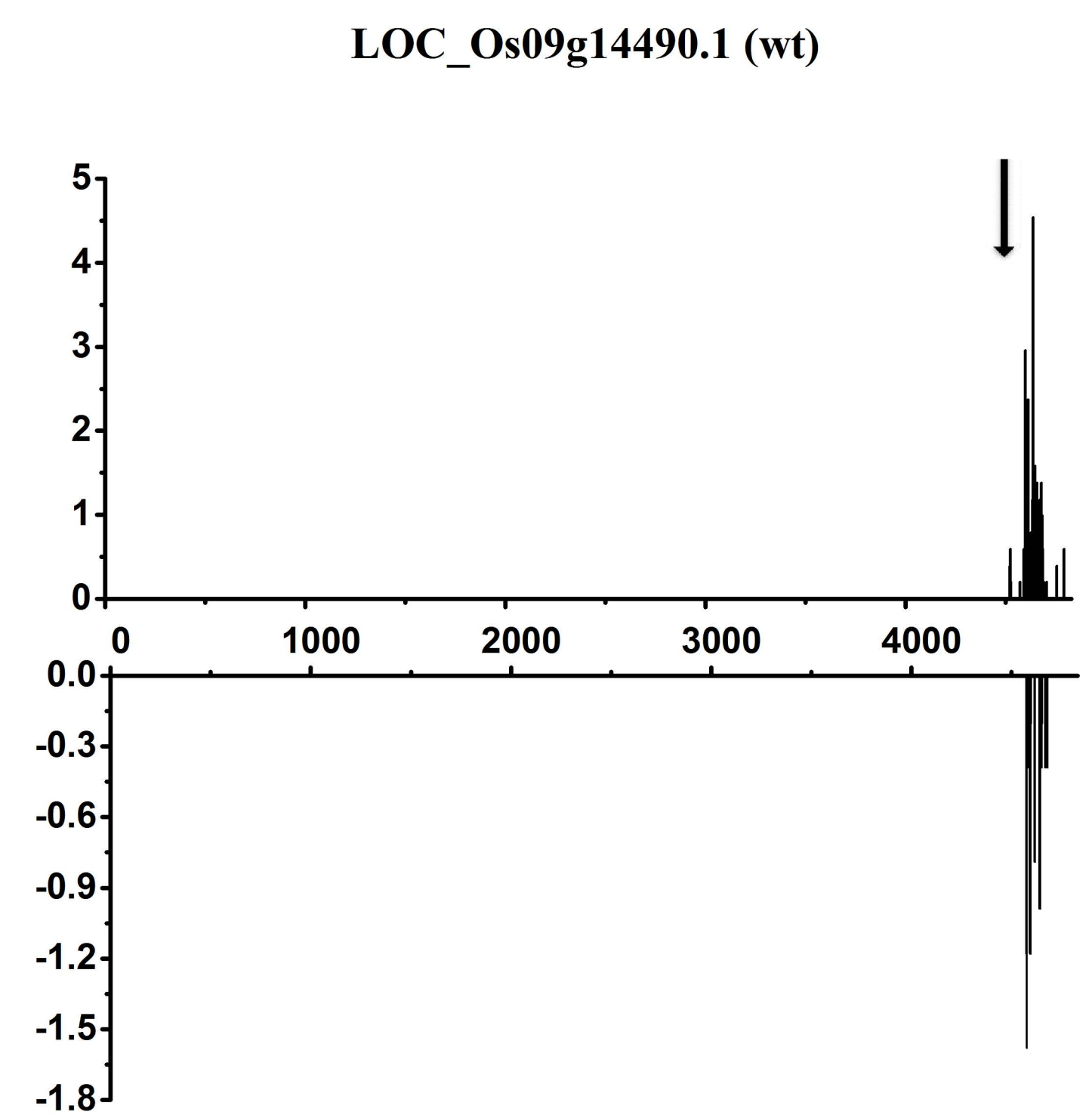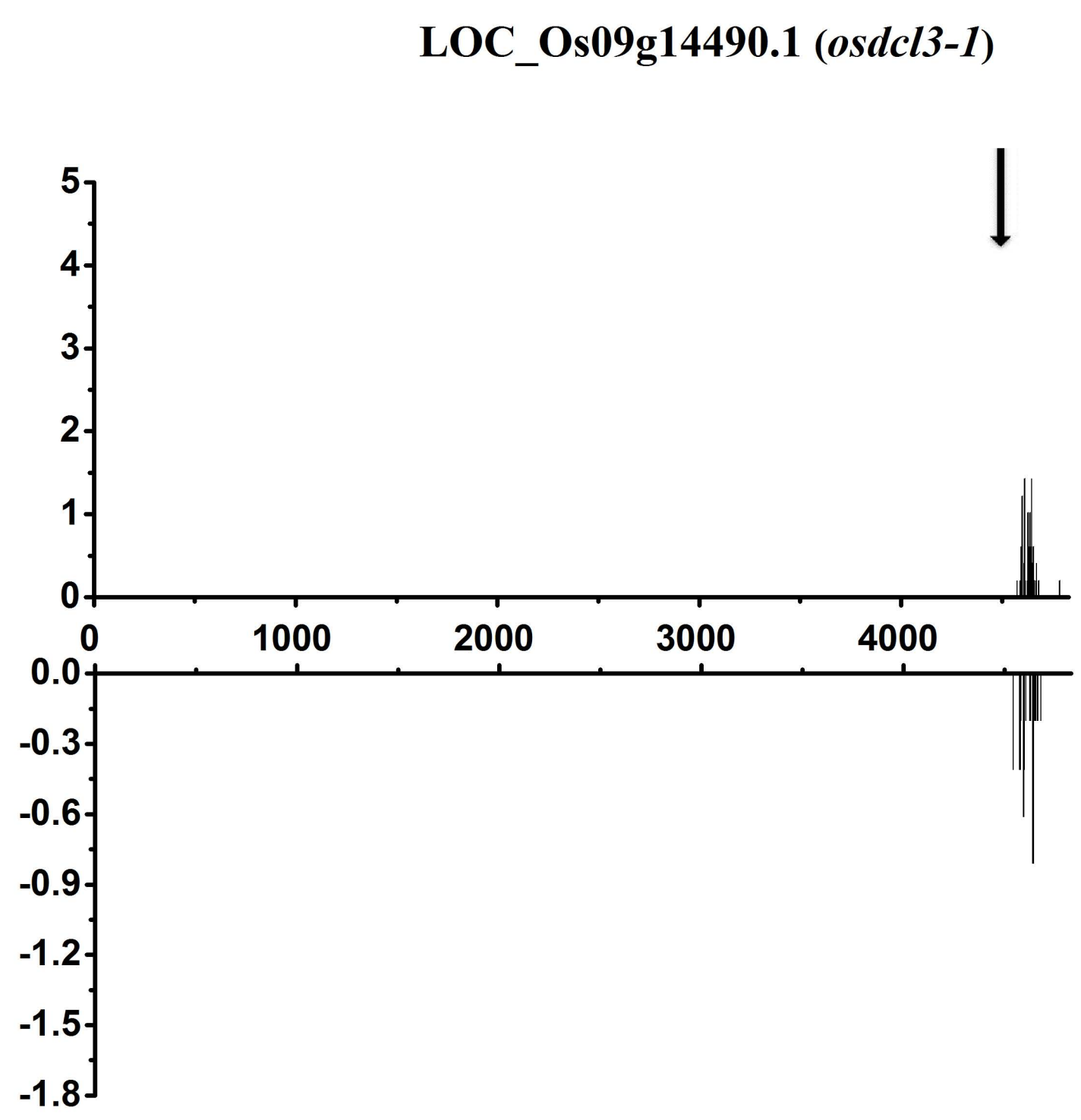

Supplement: Supplementary file 4 — Additional file 4: Figure S2. 24-nt phasiRNAs generated from the novel PHAS lociin wild-type and DCL3 mutant. 24-nt phasiRNAs generated from transcripts of LOC_Os01g37325.1 (A), LOC_Os02g20200.1 (B), LOC_Os02g55550.1 (C), LOC_Os04g45834.2 (D) and LOC_Os09g14490.1 (E) in wild-type and osdcl3–1 mutant seedling, respectively. The black arrows indicate the sRNA trigger cleavage sites, the x-axis represent the phasiRNA position mapped within the PHAS loci, the y-axis represent the read abundance (in RMP, reads per million) of the small RNAs mapped to the sense and antisense strands of PHAS loci. [file 12864_2021_7406_MOESM4_ESM.pdf]
