# Supplementary material for: Degradome sequencing-based identification of phasiRNAs biogenesis pathways in Oryza sativa
Source: BMC Genomics. 2021 Jan 30;22:93. doi: 10.1186/s12864-021-07406-7 (PMC7847607; doi:10.1186/s12864-021-07406-7)
Supplement: Supplementary file 6 — Additional file 6: Figure S3. Degradome sequencing-based validation of the phasiRNA-target interactions.Four libraries of degradome sequencing data libraries (GSM434596, GSM455938, GSM455939 and GSM476257) were recruited for T-plot profiling. The IDs of the target transcripts and the corresponding phasiRNAs generated from the transcripts of LOC_Os02g18750.1, LOC_Os05g43650.1, LOC_Os06g30680.1 and LOC_Os12g42380.1 are listed on the top. The y axis measure the normalized reads (in RMP, reads per million) of the degradome signals, and the x axis represent the position of the cleavage signals on the target transcripts. The binding sites of the phasiRNA on their target transcripts were denoted by gray horizontal lines, and the dominant cleavage signals were marked by black arrows. [file 12864_2021_7406_MOESM6_ESM.pdf]

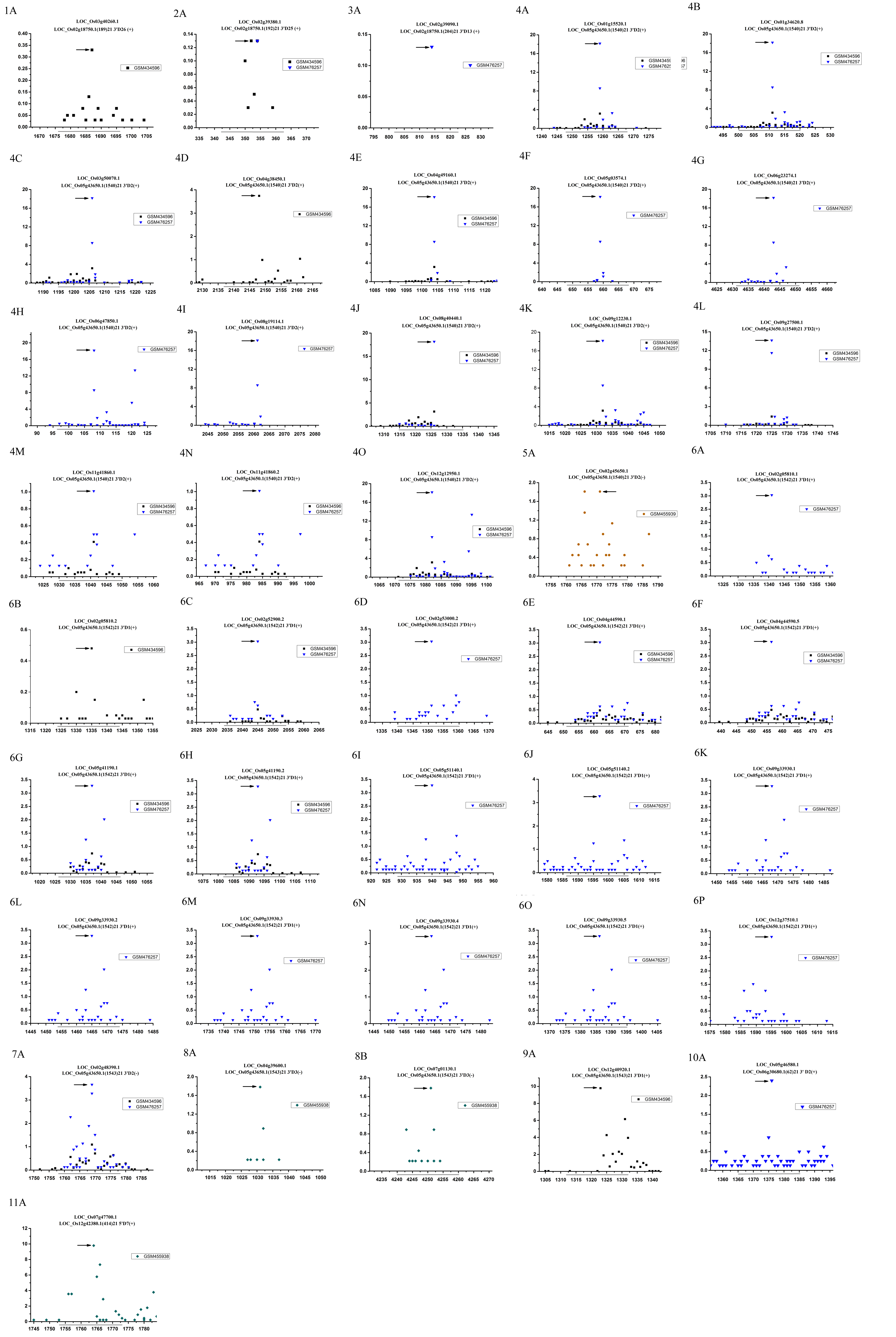
1A
LOC_Os03g40260.1
LOC_Os02g18750.1(189)21 3'D26 (+)
GSM434596
2A
LOC_Os02g39380.1
LOC_Os02g18750.1(192)21 3'D25 (+)
GSM434596
GSM476257
3A
LOC_Os02g39090.1
LOC_Os02g18750.1(204)21 3'D13 (+)
GSM476257
4A
LOC_Os01g15520.1
LOC_Os05g43650.1(1540)21 3'D2(+)
4B
LOC_Os01g34620.8
LOC_Os05g43650.1(1540)21 3'D2(+)
GSM434596
GSM476257
4C
LOC_Os03g50070.1
LOC_Os05g43650.1(1540)21 3'D2(+)
GSM434596
GSM476257
4D
LOC_Os04g38450.1
LOC_Os05g43650.1(1540)21 3'D2(+)
GSM434596
4E
LOC_Os04g49160.1
LOC_Os05g43650.1(1540)21 3'D2(+)
GSM434596
GSM476257
4F
LOC_Os05g03574.1
LOC_Os05g43650.1(1540)21 3'D2(+)
GSM476257
4G
LOC_Os06g23274.1
LOC_Os05g43650.1(1540)21 3'D2(+)
GSM476257
4H
LOC_Os06g47850.1
LOC_Os05g43650.1(1540)21 3'D2(+)
GSM476257
4I
LOC_Os08g19114.1
LOC_Os05g43650.1(1540)21 3'D2(+)
GSM476257
4J
LOC_Os08g40440.1
LOC_Os05g43650.1(1540)21 3'D2(+)
GSM434596
GSM476257
4K
LOC_Os09g12230.1
LOC_Os05g43650.1(1540)21 3'D2(+)
GSM434596
GSM476257
4L
LOC_Os09g27500.1
LOC_Os05g43650.1(1540)21 3'D2(+)
GSM434596
GSM476257
4M
LOC_Os11g41860.1
LOC_Os05g43650.1(1540)21 3'D2(+)
GSM434596
GSM476257
4N
LOC_Os11g41860.2
LOC_Os05g43650.1(1540)21 3'D2(+)
GSM434596
GSM476257
4O
LOC_Os12g12950.1
LOC_Os05g43650.1(1540)21 3'D2(+)
GSM434596
GSM476257
5A
LOC_Os02g45650.1
LOC_Os05g43650.1(1540)21 3'D2(-)
GSM455939
6A
LOC_Os02g05810.1
LOC_Os05g43650.1(1542)21 3'D1(+)
GSM476257
6B
LOC_Os02g05810.2
LOC_Os05g43650.1(1542)21 3'D1(+)
GSM434596
6C
LOC_Os02g52900.2
LOC_Os05g43650.1(1542)21 3'D1(+)
GSM434596
GSM476257
6D
LOC_Os02g53000.2
LOC_Os05g43650.1(1542)21 3'D1(+)
GSM476257
6E
LOC_Os04g44590.1
LOC_Os05g43650.1(1542)21 3'D1(+)
GSM434596
GSM476257
6F
LOC_Os04g44590.5
LOC_Os05g43650.1(1542)21 3'D1(+)
GSM434596
GSM476257
6G
LOC_Os05g41190.1
LOC_Os05g43650.1(1542)21 3'D1(+)
GSM434596
GSM476257
6H
LOC_Os05g41190.2
LOC_Os05g43650.1(1542)21 3'D1(+)
GSM434596
GSM476257
6I
LOC_Os05g51140.1
LOC_Os05g43650.1(1542)21 3'D1(+)
GSM476257
6J
LOC_Os05g51140.2
LOC_Os05g43650.1(1542)21 3'D1(+)
GSM476257
6K
LOC_Os09g33930.1
LOC_Os05g43650.1(1542)21 3'D1(+)
GSM476257
6L
LOC_Os09g33930.2
LOC_Os05g43650.1(1542)21 3'D1(+)
GSM476257
6M
LOC_Os09g33930.3
LOC_Os05g43650.1(1542)21 3'D1(+)
GSM476257
6N
LOC_Os09g33930.4
LOC_Os05g43650.1(1542)21 3'D1(+)
GSM476257
6O
LOC_Os09g33930.5
LOC_Os05g43650.1(1542)21 3'D1(+)
GSM476257
6P
LOC_Os12g37510.1
LOC_Os05g43650.1(1542)21 3'D1(+)
GSM476257
7A
LOC_Os02g48390.1
LOC_Os05g43650.1(1543)21 3'D2(-)
GSM434596
GSM476257
8A
LOC_Os04g39600.1
LOC_Os05g43650.1(1543)21 3'D3(-)
GSM455938
8B
LOC_Os07g01130.1
LOC_Os05g43650.1(1543)21 3'D3(-)
GSM455938
9A
LOC_Os12g40920.1
LOC_Os05g43650.1(1543)21 3'D1(+)
GSM434596
10A
LOC_Os05g46580.1
LOC_Os06g30680.1(62)21 3' D2(+)
GSM476257
11A
LOC_Os07g47700.1
LOC_Os12g42380.1(414)21 5'D7(+)
GSM455938
